# Development of a functionalized UV-emitting nanocomposite for the treatment of cancer using indirect photodynamic therapy

**DOI:** 10.1186/s12951-018-0344-3

**Published:** 2018-02-27

**Authors:** Prakhar Sengar, Patricia Juárez, Andrea Verdugo-Meza, Danna L. Arellano, Akhil Jain, Kanchan Chauhan, Gustavo A. Hirata, Pierrick G. J. Fournier

**Affiliations:** 1 Biomedical Innovation Department, Centro de Investigación Científica y de Educación Superior de Ensenada, Baja California, (CICESE), Carretera Tijuana Ensenada No. 3918, Zona Playitas, 22860 Ensenada, Baja California Mexico; 2 Centro de Nanociencias y Nanotecnología (CNyN), Universidad Nacional Autónoma de México (UNAM), Ensenada, Baja California Mexico; 3 Posgrado en Física de Materiales, Centro de Investigación Científica y de Educación Superior de Ensenada, Baja California, (CICESE), Ensenada, Baja California Mexico

**Keywords:** Cancer, Photodynamic therapy, X-PDT, Deep-seated tumor, Nanoscintillator, Energy transfer, ROS

## Abstract

**Background:**

Photodynamic therapy is a promising cancer therapy modality but its application for deep-seated tumor is mainly hindered by the shallow penetration of visible light. X-ray-mediated photodynamic therapy (PDT) has gained a major attention owing to the limitless penetration of X-rays. However, substantial outcomes have still not been achieved due to the low luminescence efficiency of scintillating nanoparticles and weak energy transfer to the photosensitizer. The present work describes the development of Y_2.99_Pr_0.01_Al_5_O_12_-based (YP) mesoporous silica coated nanoparticles, multifunctionalized with protoporphyrin IX (PpIX) and folic acid (YPMS@PpIX@FA) for potential application in targeted deep PDT.

**Results:**

A YP nanophosphor core was synthesized using the sol–gel method to be used as X-ray energy transducer and was then covered with a mesoporous silica layer. The luminescence analysis indicated a good spectral overlap between the PpIX and nanoscintillator at the Soret as well as Q-band region. The comparison of the emission spectra with or without PpIX showed signs of energy transfer, a prerequisite for deep PDT. In vitro studies showed the preferential uptake of the nanocomposite in cancer cells expressing the folate receptor*Folr1*, validating the targeting efficiency. Direct activation of conjugated PpIX with UVA in vitro induced ROS production causing breast and prostate cancer cell death indicating that the PpIX retained its activity after conjugation to the nanocomposite. The in vivo toxicity analysis showed the good biocompatibility and non-immunogenic response of YPMS@PpIX@FA.

**Conclusion:**

Our results indicate that YPMS@PpIX@FA nanocomposites are promising candidates for X-ray-mediated PDT of deep-seated tumors. The design of these nanoparticles allows the functionalization with exchangeable targeting ligands thus offering versatility, in order to target various cancer cells, expressing different molecular targets on their surface.

**Electronic supplementary material:**

The online version of this article (10.1186/s12951-018-0344-3) contains supplementary material, which is available to authorized users.

## Background

Cancer remains the leading cause of death in the World accounting for 8.8 million of deaths in 2015. Despite the advances in treatment that can improve patient survival or quality of life, the WHO expects that more than 14.6 millions of women and men will die from cancer in 2035, which will represent a 66% increase since 2015 [1]. It is therefore critical to develop and implement new treatment strategies such as photodynamic therapy (PDT) [2].

PDT is based on the use of photosensitizers (PS), such as molecules derived from porphyrin that are excited by photons in the UV–visible region, and then generate reactive oxygen species (ROS), usually in the form of singlet oxygen (^1^O_2_) [3]. Inside of cancer cells, these ROS oxidize proteins, lipids, and DNA causing damages leading to cell death by necrosis or apoptosis [4]. With advantages such as lack of drug resistance, selective targeting (by exciting only in the area of interest) and ease of application (for treatment of superficial tumors), PDT is a relatively less invasive alternative to conventional cancer treatments. However, due to the absorption or scattering of UV–visible photons by biomolecules present in body tissues, the current PDT suffers from poor tissue penetration and is therefore restricted to the treatment of superficial lesions [3].

Mostly in clinical practice, near infrared (NIR) light (~ 630–800 nm) is used as excitation source for treating deep cancerous tissues [5, 6]. However, the majority of clinically approved PS’s show lower absorption in the NIR window and the penetration depth of NIR for effective treatment is typically less than 1 cm [7]. Thus, various inorganic nanoparticles including quantum dots, gold nanoparticles [8, 9] and upconversion nanoparticles [10, 11], with a high extinction coefficient in NIR region are used to improve the therapeutic efficiency or alternatively, the light is delivered to deep cancerous tissues via optical fibers [7]. Although with these strategies, tissue penetration depth is achieved to some extent, new approaches are still required for treating deep-seated tumors with greater efficiency and throughout the body particularly when cancer has metastasized. Alongside NIR, X-ray as a PDT excitation source could also be therapeutically significant as it can penetrate all of our tissues, including bones [12]. However, most of the clinically approved PS’s weakly absorb direct X-ray photons and a system is needed in order to convert the X-ray into photons that can activate the PS.

Scintillating nanoparticles or nanoscintillators are energy transducers with the capability to convert X-rays into UV–visible photons. The surface of the nanoscintillators can be functionalized to deliver or carry PSs, this concept, pioneered by Chen and Zhang, has the potential to overcome the existing limitations of PDT and render possible-ray-mediated PDT (X-PDT) [13]. Subsequently, a lot more reports stated either proof of concept studies based on spectroscopic studies, or in vitro and in vivo investigations for utilization of various nanoscintillators as energy transducers for deep PDT [14–20]. Despite the encouraging progress, the X-ray induced PDT is not as efficient as conventional PDT in terms of overall ^1^O_2_ quantum yield, cancer cell toxicity and tumor inhibition [12]. The appropriate particle composition for higher radioluminescence intensity, efficient fluorescence energy transfer, and physiological stability and biocompatibility are the main properties for consideration. In general, porphyrin based PSs are used in clinical practice, which shows higher absorption in the Soret region (350–450 nm), while the majority of reported nanoparticles emits in the 450–600 nm range. This corresponds to the Q-band absorption region of porphyrin-based PS, which is less efficient in generating ROS compared to the Soret absorption region. Recently, Clement et al. reported that CeF_3_ nanoparticles emitting in the Soret region can be utilized in conjunction with most of the clinically approved PS [21]. It is known that lanthanide doped rare-earth nanoparticles possess high X-ray stopping power and also show high scintillation efficiency [12]. Thus, development of such systems with emission in Soret region could be beneficial to achieve enhanced therapeutic efficiency.

We recently demonstrated that a yttrium aluminum garnet (Y_3_Al_5_O_12_ or YAG) nanoscintillator doped with 1% praseodymium (Y_2.99_Pr_0.01_Al_5_O_12 _or YP) efficiently converts X-ray photons into UVA photons (300–450 nm) as well as in the blue (489 nm) and red (613 nm) regions [22]. Thus, YP nanoparticles represent a potential candidate for porphyrin-based X-PDT of cancer. Furthermore, stability and biocompatibility of YAG-based nanoscintillator are well suited for biomedical applications [23, 24]. On the other hand, porphyrins are mostly hydrophobic, which may limit their potential application in physiological conditions [25]. Thus, to improve the water solubility, selectivity, and stability, PSs are often incorporated into various biocompatible platforms including liposomes [26], mesoporous silica [27], microcapsules [28], polymeric micelles [27]. Mesoporous silica-coated nanoparticles have attracted much attention as PS carriers [25, 29, 30]. Silica-based NP’s hold several advantages including high surface area, pore volume, and uniform pore size. Besides the nanocarriers, such nanoparticles coated with mesoporous silica also save the loaded molecules from enzymatic degradation [31].

In the present work, we functionalized YP nanoparticles using, first, a mesoporous silica layer to obtain a robust core–shell YPMS that can be adapted with various porphyrin-based PS. We chose to use covalently conjugate protoporphyrin IX (PpIX) onto the silica layer since it has been approved for clinical use and due to its capacity to optimally generate ROS when excited with UVA photons. In addition, in order to increase the affinity of the system for cancer cells and decrease the risk of side effect in normal cells, YPMS nanoparticles were further functionalized with folic acid (FA). It has been reported that, in patients, cancer cells often express a high level of folate receptor (FOLR1) due to their metabolic needs and FA is a validated mean to target nanoparticles to cancer cells [32]. The structural properties including crystallinity, particle size, surface charge, hydrodynamic radius, mesoporous nature and organic content of the developed YPMS@PpIX@FA system were characterized. Energy transfer between the nanoscintillator and the PS was analyzed by photo- and cathodoluminescence analysis. We confirmed in vitro that YPMS@PpIX@FA nanoparticles were preferentially internalized in cells with high expression of *Folr1* and had little dark cytotoxicity. We characterized then their capability to produce ROS and kill breast and prostate cells in vitro. Finally, YPMS@PpIX@FA nanoparticles were systemically delivered in mice to assess their in vivo toxicity and their effect on the immune system. Our findings suggest that the YPMS platform could be a promising system for the treatment of deep-seated tumor using X-PDT.

## Methods

### Chemical materials

Aluminum nitrate hydrate puratronic (99.9%), yttrium (III) nitrate hexahydrate (99.9%), praseodymium (III) nitrate (99.9%) and l-tartaric acid were purchased from Alfa Aesar. All other chemicals were obtained from Sigma-Aldrich and utilized as received unless otherwise specified.

### Synthesis and optimization of mesoporous YP@SiO_2_ (YPMS) core–shell nanoparticles

The mesoporous YPMS core–shell nanoparticles were synthesized in two steps. First, the core Y_2.99_Pr_0.01_Al_5_O_12_ (YP) was synthesized using tartaric acid assisted sol–gel method as reported previously [33]. Second, the mesoporous silica layer was applied on YP using a modified version of the Stöber method [34]. Briefly, 0.15 g of synthesized YP nanoparticles were homogeneously dispersed in 100 ml of ethanol: DI water solution (4:1 v/v) using a high power 600 W ultrasonicator for 30 min. Afterward, 1 ml of ammonium hydroxide solution (28 wt%) was added, followed by dropwise addition of 24 µl of tetraethyl orthosilicate (TEOS). The mixture was then incubated at room temperature, for 6 h, under constant stirring. The product was collected by centrifugation (3000 rpm, 10 min), washed first with DI water and then with ethanol.

In order to optimize the mesoporous layer on the core–shell, first, the washed nanoparticles were re-suspended in 140 ml of an ethanol: DI water solution (3:4 v/v), containing 0.3 g of cetyl trimethyl ammonium bromide (CTAB) and 1.0 ml of ammonium hydroxide solution (28 wt%). The suspension was then homogenized under constant stirring for 30 min. Different volumes of TEOS (60, 150 and 250 µl) were then added to the reaction mixture. After 8 h, the product was collected by centrifugation and washed with ethanol and then with water. The as-synthesized materials were dried at 80 °C for 8 h and annealed in air at 550 °C for 5 h to make the silica layer crystalline.

### Surface modification of YPMS

To use YPMS nanoparticles for PDT application, the surface of the construct was further modified by covalent conjugation of folic acid (FA, targeting agent) and protoporphyrin IX (PpIX, photosensitizer). The carboxylic group present in PpIX and FA were first conjugated with the amino group of aminopropyltrimethoxysilane (APTMS) through EDC–NHS coupling. After that, the modified APTMS was conjugated onto the mesoporous silica at the surface of YPMS, by hydrolysis of methoxy group of APTMS followed by the adsorption of hydroxy silane product forming covalent –Si–O–Si– linkage. PpIX (0.006 mmol), FA (0.002 mmol) and APTMS (20 µl) were dissolved in 5 ml DMSO and then EDC (0.012 mmol) and NHS (0.028 mmol) were added. The reaction mixture was stirred for 2 h at room temperature. Meanwhile, YPMS (50 mg) was suspended in 3 ml of DMSO by sonication, for 30 min. The YPMS suspension was then added dropwise to the reaction mixture and left overnight under constant stirring at room temperature. The final product was collected by centrifugation (3000 rpm, 10 min) and washed thrice with DMSO and then with ethanol, to remove the residual precursors. Finally, the surface modified nanoparticles were dried at 80 °C for 8 h.

The concentration of PpIX on YPMS@PpIX@FA was determined using the characteristic absorption of PpIX at 405 nm, assuming there was no change in the extinction coefficient of PpIX. A calibration curve was obtained by recording the UV–Vis absorption spectrum of varying concentrations of PpIX in DMSO, using a Cary 60 UV–Vis spectrophotometer (Agilent). Afterward, the spectrum of YPMS@PpIX@FA in DMSO was recorded, and the final concentration of PpIX on nanoparticle surface was determined.

### Characterization of nanoparticles

The morphology of the nanoparticles and the thickness of the mesoporous silica layer were analyzed by means of a transmission electron microscope (TEM, JEOL-2010, JEOL) operated at 200 kV. The thickness of the mesoporous silica layer of 50 different nanoparticles was measured using the Gatan Microscopy Suite Software (v2). The crystalline phases of the postannealed powders were identified by X-ray diffraction (XRD, Philips X’pert diffractometer) using CuK_αI_ (λ = 1.54 Å) radiation at a scanning rate of 0.5 degrees/min. Dynamic light scattering (DLS) and Zeta-potential measurements were conducted on Zetasizer Nanoseries (Nano-ZS, Malvern Instruments). Nitrogen adsorption–desorption isotherms were evaluated on a Micromeritics TriStar II (Micromeritics Instrument Ltd.). The specific area was calculated using the Brunauer–Emmett–Teller (BET) method. Barrerr–Joyner–Halanda (BJH) method was utilized to derive pore-size distribution. The surface modifications on the nanoparticles were analyzed by Fourier-Transform Infrared Spectroscopy (FTIR) (Nicolet 5700 FT-IR spectrometer). The photoluminescence (PL) spectra measurements were conducted at room temperature in a Hitachi fluorescence spectrometer F-7000equipped with a 150 W Xe-lamp as the excitation source. The cathodoluminescence (CL) measurements in Gatan mono-CL system in UV–Vis range coupled with scanning electron microscope (JSM-7800F, JEOL). X-ray photoelectron spectroscopy (XPS) measurements were done in a SPECS system equipped with a PHOIBOS WAL analyzer using Al anode. The UV–Vis absorption spectra of various samples were measured on a Cary 60 UV–Vis spectrophotometer.

### DPBF assay

To detect singlet oxygen in solution, we used 1,3-diphenylisobenzofuran (DPBF) as a singlet oxygen sensor [35]. PpIX or YPMS and YPMS@PpIX@FA nanoparticles were dissolved or suspended in DMSO using a high power 600 W ultrasonicator for 30 min. Sonication was done to decrease the light scattering by nanoparticles in the samples as it is very sensitive to the aggregation of the particles [36]. The optical density at 405 nm of the solutions containing PpIX was measured in quartz cuvettes and adjusted to 0.8, in order to have a similar concentration of PS, in suspension or bound to the YPMS nanocomposite. DPBF (Sigma-Aldrich) was dissolved in DMSO at 8 mM extemporaneously and added to the samples to obtain a final concentration of 64 μM, in a total volume of 1 ml. The reaction mixture was irradiated with a UV_254 nm_ lamp (UVL-28 EL, UVP, USA) for different time intervals (0–120 s) in plastic cuvettes. The DPBF fluorescence was measured at λ_em_ = 485 nm with an excitation at λ_ex_ = 410 nm. The experiment was performed in triplets in the set of three individual experiments.

### Cell culture

Mouse breast cancer cell lines 4T1, prostate cancer cell lines TRAMP-C1 and TRAMP-C2 and the melanoma cell line B16-F1 were obtained from the American Type Culture Collection (ATCC). PyMT-R221A mouse breast cancer cell line was obtained from Dr. Conor Lynch (Moffit Cancer Center), and RM-1 mouse prostate cancer cell line was obtained from Dr. Timothy Thompson (The University of Texas, MD Anderson Cancer Center). PyMT-R221A and TRAMP-C2 were cultured in high-glucose DMEM media (Corning) supplemented with 10% heat-inactivated fetal bovine serum (FBS, Biowest) and 1% antibiotic/antimycotic solution (Corning). 4T1 and RM-1 were cultured in RPMI media (Corning) supplemented with 10% heat-inactivated FBS and 1% antibiotic/antimycotic solution. TRAMP-C1 and B16-F1 were cultured in RPMI supplemented with 5% heat-inactivated FBS and 1% antibiotic/antimycotic solution. Cells were maintained at 37 °C, with 5% CO_2_ in a humidified incubator. To generate PyMT-R221A expressing ß-galactosidase, PyMT-ßGal, the coding sequence of LacZ was subcloned from the pcDNA 3.1 Zeo LacZ (Invitrogen) into the lentiviral vector pLJM1, a gift from David Sabatini (Addgene plasmid # 19319) [37] using the Gibson Assembly Cloning kit (New England Biolabs). Lentiviral particles were then generated in 293T cells and collected to transduce PyMT-R221A. Transduced cells were then selected by culturing them in puromycin. Expression of LacZ in PyMT-ßGal was confirmed performing a modified version of the ß-Gal assay kit (Invitrogen). Briefly, 39 µl of cell culture supernatant (cleared of debris by centrifugation) were mixed with 194 µl of cleavage buffer (1×) containing-mercaptoethanol and 66 µl of ONPG (8 mg/ml). After 2 h at 37 °C, the optical density at 420 nm was measured.

### RNA isolation and quantitative RT-PCR

Total RNA was extracted from cultured cell lines using GenElute™ Mammalian Total RNA Kit (Sigma-Aldrich) according to manufacturer’s instructions. RNA was quantified using a NanoDrop Lite (Thermo Fisher Scientific), and 250 ng were reverse-transcribed using anchored oligodT primers (Thermo Fisher Scientific) and Superscript II reverse transcriptase (Thermo Fisher Scientific) as per the company’s instructions. cDNA was analyzed in triplicate by quantitative real-time PCR using HotStart-IT SYBR Green PCR master kit (USB Affymetrix) for 40 cycles (95 °C for 15 s, 58 °C for 30 s and 72 °C for 30 s). Primers were designed using Primer3Plus [38] and purchased from T_4_ Oligo. Primer sequences used were: *Rpl32* (sense, 5′-CAGGGTGCGGAGAAGGTTCAAGGG-3′; antisense, 5′-CTTAGAGGACACGTTGTGAGCAATC-3′) and *Folr1* (sense, 5′-ATGAGTGTTCCCCGAACTTG-3′; antisense, 5′-ACACAGAGCAGCAGATGTGG-3′).

Quantification of folate receptor 1 (*Folr1*) gene expression was performed using standard curves of diluted cDNA templates, and relative amounts were normalized to the housekeeping gene ribosomal protein L32 (*Rpl32*).

### In vitro cellular uptake studies

Fifty thousand TRAMP-C1 or RM-1 cells were seeded in 35 mm glass bottom dishes (MatTek Co.). After 24 h, cells were treated with YPMS@PpIX@FA (50 µg/ml) for 30 or 180 min. Cells were then washed three times with PBS, fixed with 4% paraformaldehyde and counterstained with DAPI. The cells were then analyzed using an inverted laser scanning microscope FV1000 FluoView (Olympus) equipped with ʎ_ex_ = 405 nm and ʎ_em_ = 430–470 nm for DAPI and ʎ_ex_ = 543 nm and ʎ_em_ = 655–755 nm for PpIX. Images were captured using FluoView software (Olympus).

### In vitro cytotoxicity analysis

To assess the toxicity of non-activated nanoparticles or dark toxicity, TRAMP-C1, RM-1, 4T1, and PyMT-R221A cells were seeded in tissue culture treated 96-well plates (4000 cells/100 µl per well) and incubated for 24 h. Cells were then treated with YPMS or YPMS@PpIX@FA at varying concentrations (6.25, 12.5, 25, 50 or 100 µg/ml) and further incubated for 24 or 48 h. Cell viability was assessed using the MTT method. Briefly, 20 µl of MTT (5 mg/ml in PBS) was added, and the plate was further incubated for 4 h. MTT-formazan formation was stopped by adding 100 µl of SDS (10%) solution in HCl (0.01 M). Plates were kept for 18 h at 37 °C, and optical density was measured at 570 nm using an Epoch plate reader (BioTek). Experiments were run in quadruplicate, and 3 independent experiments were performed. To assess the phototoxicity or toxicity of activated YPMS and YPMS@PpIX@FA, TRAMP-C1, PyMT-R221A and PyMT-ß-Gal cells were seeded as previously and incubated for 24 h. Cells were then treated with increased concentration of YPMS or YPMS@PpIX@FA (6.25, 12.5, 25, 50 or 100 µg/ml). To assess the phototoxic effect of all the nanoparticles (in solution and internalized), the cells were cultured for 24 h before being exposed or not exposed to increasing doses of UV light at 365 nm (from 0.1 to 3 J/cm^2^), using a CL-508 UV Crosslinker (Cleaver Scientific Ltd.). UV doses were monitored using a UV radiometer (VLX-3 W, VilberLourmat). The cells were then incubated for 24 h before measuring cells viability using the MTT method or cell death using a ß-galactosidase release assay. To assess the phototoxic effect only of the internalized nanoparticles, the media was removed after 24 h of treatment and the cells washed with PBS, to eliminate non-internalized nanoparticles. Cells were then exposed or not exposed to UV and further cultured for 24 h before measuring cell viability. For ß-galactosidase release assay, cell culture supernatant of PyMT-ßGal cells was collected and centrifuged to eliminate cell debris. ß-Galactosidase was then measured as described previously.

### ROS detection

PyMT-R221A or TRAMP-C1 cells were seeded in tissue culture treated 6-well plates (80,000 cells/2 ml per well) and incubated for 24 h. Cells were then treated with YPMS@PpIX@FA (6.25, 12.5 or 25 µg/ml) and further cultured for 24 h. Cells were then exposed to UV_365 nm_ (0, 125, 250 or 500 mJ/cm^2^) and 100 µl of 2′,7′-dichlorofluorescindiacetate (DCFDA, 800 µM) was added to the culture. After 30 min of incubation, cells were trypsinized and analyzed using an attune acoustic focusing cytometer (Thermo Fisher Scientific). Three independent experiments were carried out, and 10,000 events were collected for each sample. Attune Cytometric Software (v2.1) was used to analyze results.

### Animal experiments

All animal experiments were performed in compliance with the local ethics committee of the CICESE. Male CD-1 mice (8-week old) were obtained from Harlan–Envigo. Mice were maintained in an Optimice cage system (Animal Care Systems), in a controlled environment room (temperature 24 °C and 12 h light/dark cycle) [39]. Mice received water and food (2018 Teklad Global 18% protein rodent diet) ad libitum. Mice were acclimated for at least a week before starting the experiments.

### Acute and sub-acute toxicity study of YPMS@PpIX@FA in mice

*Acute toxicity study* CD-1 mice (n = 5 per group) were observed for a week of the adaptive period during which food intake, water intake and weight was monitored 3 times per week. After this adaptive period, mice were inoculated once in the tail vein with 100 µl of PBS or YPMS@PpIX@FA suspension in PBS (0, 62.5, 125, 250 and 500 mg/kg), using a 1 ml syringe with a 29G needle. After injection, we kept on recording food and water intake and body weight 3 times per week. Mice were monitored daily for signs of discomfort and were either euthanized 7 days after inoculation or when presenting evidence of distress. Mice were euthanized in a CO_2_ chamber, and cervical dislocation was used as a secondary method of euthanasia.

*Sub*-*acute toxicity study*: CD-1 mice were inoculated in the tail vein with 100 µl of PBS (n = 6) or of a YPMS@PpIX@FA suspension (5 and 25 mg/kg, n = 7 per group), 3 times per week, for 2 weeks, using a 1 ml syringe with a 29G needle. At the end of the treatment period, 50 µl of blood was collected from the retro-orbital sinus of the mice using heparinized blood collection capillaries to analyze peripheral blood mononuclear cells using flow cytometry. Mice were further observed for 2 weeks. Food and water intake, as well as body weight, were recorded 3 times per week throughout the protocol. Mice were monitored daily for signs of discomfort. Mice were euthanized in a CO_2_ chamber, and cervical dislocation was used as a secondary method of euthanasia. Organs were collected and fixed for 24 h in a formalin buffered solution (4 °C). Tissues were processed in a TP1020 Semi-enclosed Benchtop Tissue Processor (Leica) and embedded in paraffin wax for sectioning. Sections (8 µm thickness) were cut using an RM2245 Semi-Automated Rotary Microtome (Leica) and stained with haematoxylin and eosin (H&E). Images were collected using a BX61 microscope (Olympus), and histological assessment of tissues was done using the standard technique.

### Flow cytometry analysis of mouse peripheral blood mononuclear cells (PBMCs)

Fifty microliters of peripheral blood were collected from the retro-orbital sinus of mice. Red blood cells were lysed with red blood cell lysis buffer (BioLegend). Cells were labeled with antibodies against CD3ε-APC (145-2C11; eBioscience), CD4-PE-Cy5.5 (RM4–5; eBioscience), CD8a-AlexaFluor 488 (53–6.7; BioLegend) and CD69-PE (H1.2F3; eBioscience) or against CD11b-FITC (M1/70; BioLegend) in a blocking buffer containing an anti-CD16/CD32 antibody (93; eBioscience) to block Fc receptors. Cells were analyzed using an attune acoustic focusing cytometer (Thermo Fisher Scientific). Cell doublets were gated out using FSC-A vs. FSC-H, and SSC-A vs. SSC-H density plots and PBMCs were gated using FSC-A vs. SSC-A. At least 30,000 events in the PBMC gate were collected. Attune Cytometric Software (v2.1) was used to analyze results.

### Statistical analysis

Statistical analyses were performed using GraphPad Prism v7.0 software (GraphPad Software, Inc.). Comparisons of three or more groups were conducted with a 1-way ANOVA test, followed by a Tukey post-test. For responses that were affected by two variables, a 2-way ANOVA with a Tukey post-test was used. Results are expressed as mean ± SEM and a p ≤ 0.05 was considered significant.

## Results

### Preparation and characterization of YPMS@PpIX@FA nanoparticles

**Scheme 1:**
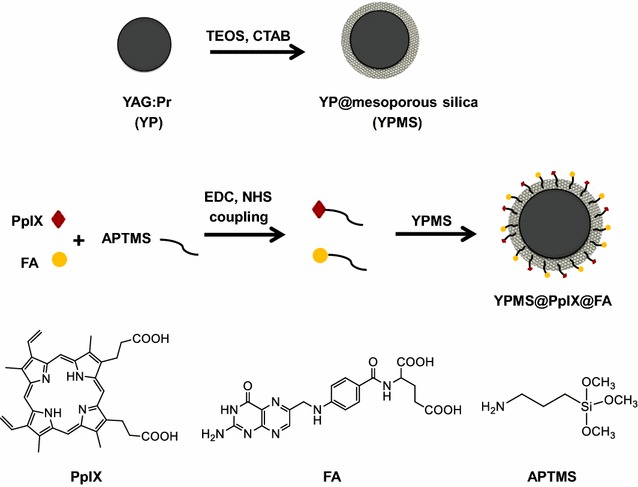
Schematic representation for the synthesis of YPMS@PpIX@FA nanocomposite

**Fig. 1:**
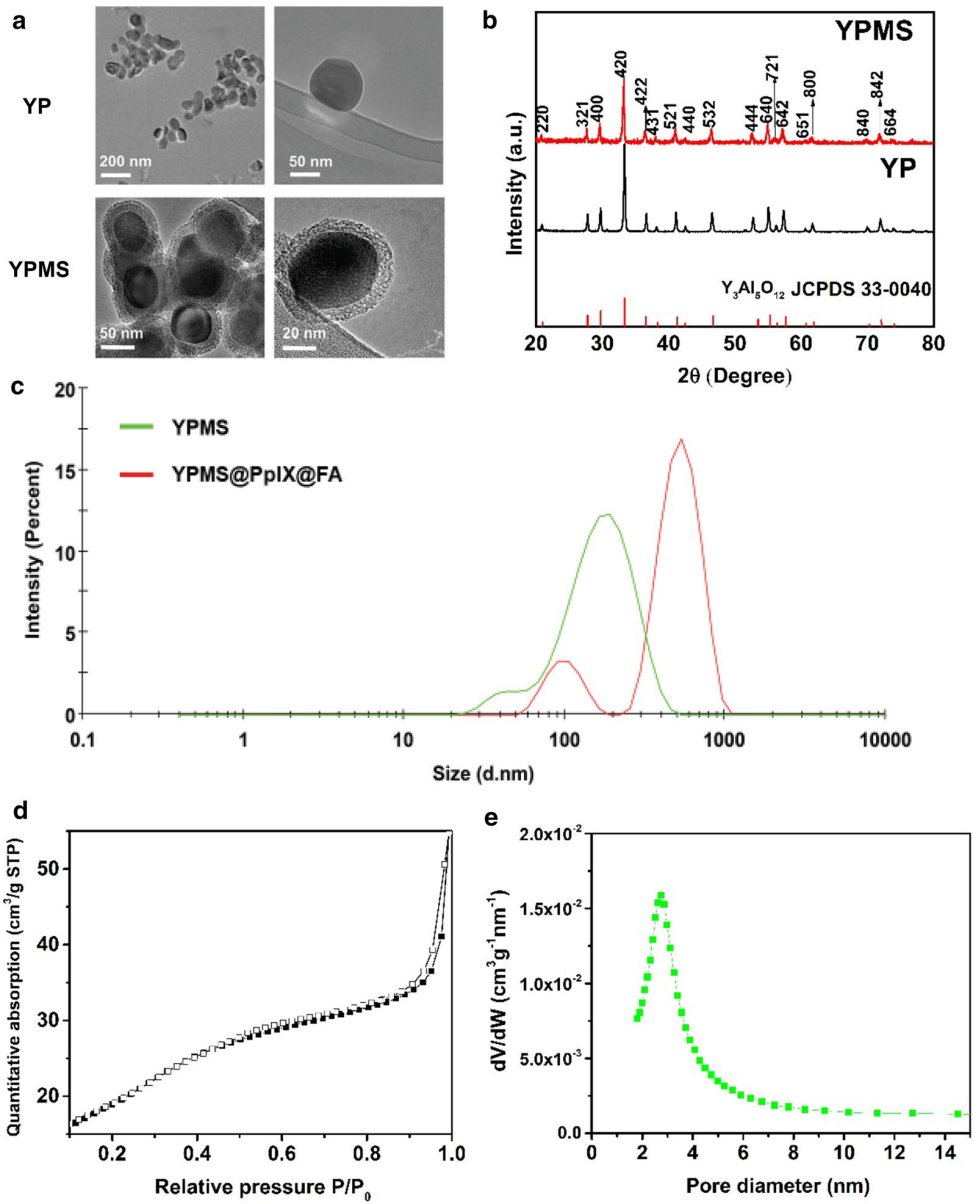
Morphological characterization YPMS nanoparticles. **a** Transmission electron microscopy images of **a** bare YP and YP nanoparticles covered with mesoporous silica (YPMS). **b** X-ray diffraction pattern of YP bare or covered with mesoporous silica, YPMS. Values correspond to hkl coordinate of the YAG cubic crystal. **c** Hydrodynamic diameters of YPMS and YPMS@PpIX@FA in water using dynamic light scattering. **d** Nitrogen adsorption–desorption isotherms of YPMS nanoparticles. Filled square adsorption, empty square desorption. **e** Pore size distribution of the mesoporous layer of YPMS nanoparticles

**Fig. 2:**
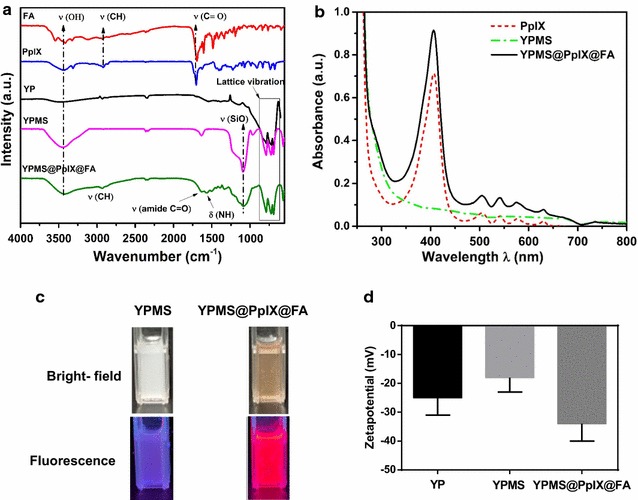
Functionalization of YPMS nanoparticles with PpIX and folic acid. **a** FTIR spectra of folic acid (FA), protoporphyrin IX (PpIX), YP, YPMS and YPMS@PpIX@FA nanoparticles. **b** UV–Vis spectra of PpIX (1.75 µg/ml), YPMS (100 µg/ml), and YPMS@PpIX@FA (25 µg/ml) nanoparticles with equivalent concentration of PpIX (~ 2 µg/ml). **c** Bright-field and fluorescence images (λ_ex_ = 365 nm) of YPMS and YPMS@PpIX@FA nanoparticles. **d** Zeta potential variation of YP, YPMS and YPMS@PpIX@FA nanoparticles in DI water

The YPMS@PpIX@FA nanoparticles were synthesized in three steps (Scheme 1). First, YP spherical cores with a 75 nm average diameter were produced using the sol–gel method [22, 33]. YP was selected based on its radioluminescence emission properties in the 300–450 nm range, which is critical for the activation of the photosensitizer protoporphyrin IX (PpIX). A mesoporous silica shell (MS) was then coated on the surface of the YP core, which was confirmed by transmission electron microscopy (TEM) (Fig. 1a and Additional file 1: Figure S1a). X-ray diffraction pattern revealed sharp and distinct peaks confirming that the YP core retained its crystallinity after the mesoporous silica coating (Fig. 1b). In addition, the cubic phase of YP uncoated or coated with silica correlated with the phase of pure cubic YAG (JCPDS file:33–40) (Fig. 1b). To optimize the porous silica layer, various concentrations of TEOS were tested. Using final concentrations higher than 0.1% (v/v) led to the formation of shells too thick for the biomedical application (20–50 nm) as well as resulted in self-nucleation of silica (Additional file 1: Figure S1a). A TEOS concentration of 0.04% (v/v) allowed the formation of a homogenous, 14 ± 4 nm thick layer (Fig. 1a and Additional file 1: Figure S1b), increasing the average size of the nanoparticles to more than 100 nm as confirmed by TEM. Dynamic light scattering (DLS) analysis indicated that, in aqueous solution, the YPMS nanoparticles had a Z-average diameter of 177 nm (PdI: 0.36) (Fig. 1c). This value, higher than the TEM-diameter is probably due to the hydration of the silica layer. To further characterize the silica layer, we analyzed nitrogen adsorption and desorption on the nanoparticles. Using the BET model, we calculated that YP nanoparticles with the silica layer had a surface area of 70 m^2^/g, 10-fold more than the area of the core YP nanoparticle, 7 m^2^/g [33], confirming the formation of a porous layer. Type IV isotherms with H1 hysteresis loops indicated the presence of mesopore, with a high uniformity of pore size (Fig. 1d). Furthermore, the BJH model indicated an average pore diameter of 3.8 nm, with narrow distribution (Fig. 1e), confirming the synthesis of YP nanoparticle with a mesoporous silica layer (YPMS). Finally, the surface of YPMS nanoparticles was covalently conjugated with PpIX, as PS, and folic acid (FA), as a targeting agent for cancer cells, using APTMS as a linker (Scheme 1). The surface modifications with PpIX and FA increased the hydrodynamic diameter of particles to ~ 747 nm (PDI: 0.5) (Fig. 1c), a phenomenon also observed in the literature, probably due to the aggregation of hydrophobic PpIX-conjugated particles [29]. FTIR spectra were used to confirm the functionalization of the nanoparticles. YP and YPMS nanoparticles exhibited vibrational peaks at lower frequencies (456, 692, 724 and 788 cm^−1^) consistent with the metal–oxygen, Y–O, and Al–O vibrations from the core YAG crystal lattice of the nanoparticle (Fig. 2a). After addition of the mesoporous layer, we detected new asymmetric vibrations with a peak at 1110 cm^−1^, characteristic of Si–O–Si, which confirmed the silica coating (Fig. 2a). After conjugation of PpIX and FA on the YPMS nanoparticles, the appearance of a CH_2_ peak stretching at 2929 cm^−1^ in YPMS@PpIX@FA indicated the presence of FA and PpIX as they contain similar absorption peaks. In addition, the peak at 1700 cm^−1^, characteristic of C=O bond from carboxylic functions of PpIX and FA shifted to a lower vibration frequency at 1641 cm^−1^ characteristic of the amide function. A new peak at 1558 cm^−1^ further confirmed the formation of an amide (NH–C=O) function during the conjugation (Fig. 2a). These changes in the FTIR spectra demonstrate the covalent binding of FA and PpIX to the YPMS nanoparticles via an amide bond. Accordingly, analysis of UV–Vis absorption spectra confirmed the presence of a broad absorption peak at ~ 410 nm on the YPMS@PpIX@FA nanoparticles as well as a series of absorption peaks between 500 and 650 nm corresponding to the Soret band and the Q-bands, respectively (Fig. 2b). These peaks are characteristics of PpIX and are absent in the YPMS spectrum (Fig. 2b). UV–Vis absorption spectrum was also used to estimate the concentration of PpIX loaded on YPMS@PpIX@FA, which was calculated as 0.144 µmol of PpIX per mg of material. As UV–Vis absorption range of FA falls in the absorption region of YPMS and PpIX, therefore, a distinct peak of folic acid was not visible in the absorption spectrum of YPMS@PpIX@FA for FA quantification (Additional file 1: Figure S2a). Observation of YPMS@PpIX@FA nanoparticles under UV light at 365 nm showed a bright red fluorescence that was not visible with YPMS nanoparticles and is due to the functionalization with PpIX (Fig. 2c). Finally, zeta potential measurements of the different nanoparticles in deionized water showed variations in the zeta potential of core–shell YPMS (− 18 ± 5 mV), and YPMS@PpIX@FA (− 34 ± 6 mV) suggesting the successful modification of the particle surface (Fig. 2d). In addition, this highly negative charge on YPMS@PpIX@FA confirms that there is a negligible risk of attraction between the nanoparticles, resulting in a better dispersity in solution. Overall these data demonstrate that we synthesized YP core nanoparticles coated with a mesoporous silica layer that was successfully functionalized with the photosensitizer PpIX and the targeting agent FA.

### Photophysical characterization

**Fig. 3:**
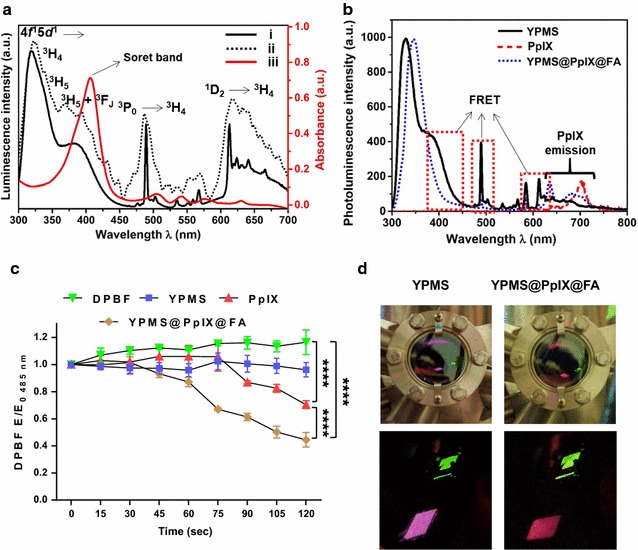
Photophysical properties of YPMS@PpIX@FA functionalized nanoparticles. **a** Overlay of YPMS nanoparticles [i] photoluminescence spectrum at λ_ex_ = 290 nm, [ii] cathodoluminescence spectrum at 15 keV, and [iii] absorption spectrum of PpIX. **b** Photoluminescence spectra of PpIX, YPMS and YPMS@PpIX@FA nanoparticles in DMSO, at λ_ex_ = 290 nm. **c** Photo-oxygenation of DPBF after exposition to UV light (254 nm) in the presence of YPMs, free PpIX or PpIX conjugated to YPMS (YPMS@PpIX@FA). Results are the mean ± SEM ratio of the fluorescence extinction (F/F0) at 485 nm. ****p < 0.0001 using a 2-way ANOVA with Tukey posttest. **d** Image of YPMS and YPMS@PpIX@FA nanoparticles on exposure of X-ray of 1.48 keV

The photoluminescence (PL) spectrum of powdered YPMS was measured at room temperature, at the excitation wavelength of 290 nm as it corresponds to the highest peak in the excitation spectrum of YP nanoscintillator [33]. The PL spectrum is comprised of a broad PL band from 300 to 450 nm that corresponds to the Pr^3+^ transition from a 4f^1^5d^1^ state → 4f^2^ (^3^H_4_, ^3^H_5_, ^3^H_6_ + ^3^f_J_) states (Fig. 3a). Additionally, the sharp PL peaks around 489 and 613 nm correspond to ^3^P_0_ → ^3^H_4_ and ^1^D_2_ → ^3^H_4_ f–f transitions of Pr^3+^ (Fig. 3a). It is important to note that YPMS give the strongest emission in 300–450 nm (UVA), which lies in the range of maximum absorbance of PpIX. It is already known that the phototoxicity of PpIX induced by UVA is superior to the one induced by visible light leading to improved PDT efficiency [40]. There was a good spectral overlap between YPMS emission spectrum and PpIX absorption spectrum in Soret as well as Q-band (Fig. 3b), suggesting that effective energy transfer is possible in most of the absorption regions of PpIX, which may result in highly efficient deep PDT response. Also, the measurement of the cathodoluminescence spectrum of YPMS nanoparticles obtained by high-energy electron bombardment revealed a spectrum similar to the one obtained by UV excitation (Fig. 3a). Noticeably, similar luminescence spectrum for YP was also obtained under X-ray irradiation in our previous work [22]. Thus, UV light could be used as an excitation source to compare the luminescence properties of YPMS and YPMS@PpIX@FA.

We compared then the PL emission spectra of YPMS and YPMS@PpIX@FA nanoparticles and free PpIX (1 mg/ml) in DMSO after excitation at 290 nm. YPMS showed the characteristic emission spectrum of YP (Fig. 3b). Functionalization with PpIX led to quenching of YPMS emission in the Soret region (~ 410 nm) as well as in the Q-band region, and the new emission peaks centered at ~ 635 and ~ 700 nm, characteristics of PpIX emission (Fig. 3b). These modifications clearly indicate the presence of energy transfer between the core YP nanoparticles and PpIX molecules. For most of the reported X-PDT nanocomposites utilizing porphyrin-based PS, FRET was achieved only in the Q-band region [41]. Remarkably in the developed system, energy transfer was not only possible in the Q-band region but also in the Soret region of PpIX, it should then result in a more efficient energy transfer from the YP core to PpIX. To assess the capability of the functionalized nanoparticles to generate singlet oxygen, a type of ROS, in solution, we used 1,3-diphenylisobenzofuran (DPBF). The irreversible chemical reaction of DPBF with singlet oxygen causes a decrease of its fluorescence intensity. We analyzed the capability of PpIX, YPMS or YPMS@PpIX@FA to oxidize DPBF when exposed to UV light (254 nm), one of the excitation wavelength of YP (Additional file 1: Figure S2b). This range was chosen due to the low absorption of PpIX at 254 nm and easy availability of UV_254 nm_ lamp. The YPMS nanocomposite in solution did not cause the photo-oxygenation of DPBF when compared to the control solution (Fig. 3c). PpIX alone, in solution, was able to decrease the emission of DPBF. However, PpIX conjugated YPMS nanocomposite significantly increased the degradation of DPBF compared to PpIX alone, at similar concentrations (Fig. 3c). This increased photo-oxygenation of DPBF suggests a more effective production of ROS when PpIX is coupled to YPMS, presumably due to the energy transfer from the YP core to the conjugated PpIX. The X-ray-mediated energy transfer was also verified by visualization of YPMS and YPMS@PpIX@FA emission after X-ray irradiation (1.48 keV). As expected, YPMS showed characteristic violet emission of YP core whereas YPMS@PpIX@FA exhibited red emission due to surface functionalized PpIX (Fig. 3d) further illustrating that the efficient energy transfer could be achieved with the developed nano-PDT platform.

### In vitro targeting and internalization of YPMS@PpIX@FA

**Fig. 4:**
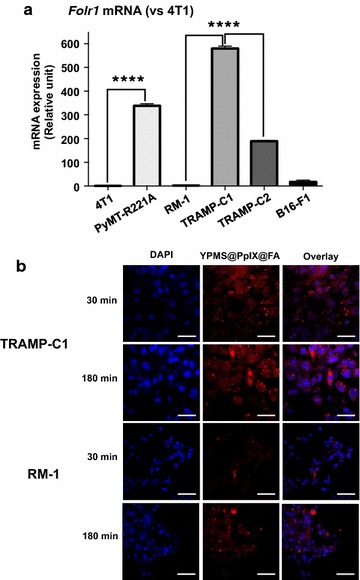
Preferential uptake of YPMS@PpIX@FA in TRAMP-C1 cells. **a** Analysis of *Folr1* mRNA levels in various mouse cancer cell lines. Results are expressed as the mean ± SEM (n = 3). ****p < 0.0001 using a 1-way ANOVA with a Tukey posttest. **b** Representative confocal fluorescence images of TRAMP-C1 and RM-1 cells treated for 30 or 180 min with YPMS@PpIX@FA nanoparticles and counterstained with DAPI. Scale bar, 50 µm

Folate receptor α (*FOLR1*) is often overexpressed in cancer cells of patients and folic acid (FA) has been used to increase the affinity of a variety of anti-cancer agents for cancer cells [42]. To test the functionalization of the YPMS@PpIX@FA nanoparticles, we screened various mouse cancer cell lines to identify cells with high and low expression of *Folr1* mRNA. Among the mouse breast cancer cell lines tested, PyMT-R221A had 338-times more *Folr1* mRNA than 4T1 cells (Fig. 4a). In prostate cancer cells, *Folr1* mRNA expression was 329- and 107-times higher in TRAMP-C1 and TRAMP-C2 cells, respectively, when compared to RM-1 cells (Fig. 4a). We used these results to compare the effect of YPMS@PpIX@FA between *Folr1*^*hi*^ cells (PyMT-R221A and TRAMP-C1) and *Folr1*^*lo*^ cells (4T1 and RM-1).

To study the uptake of the nanoparticles, TRAMP-C1 and RM-1 cells were cultured in the presence of YPMS@PpIX@FA before analyzing the cells using confocal microscopy. The red fluorescence of PpIX was stronger in *Folr1*^*hi*^ TRAMP-C1 cells when compared to *Folr1*^*lo*^ RM-1 cells after for 30 or 180 min of incubation (Fig. 4b). This result shows that functionalization of YPMS nanoparticles with FA allows a better uptake in cancer cells with higher expression of folate receptor.

### In vitro cyto- and phototoxicity analysis of YPMS@PpIX@FA

**Fig. 5:**
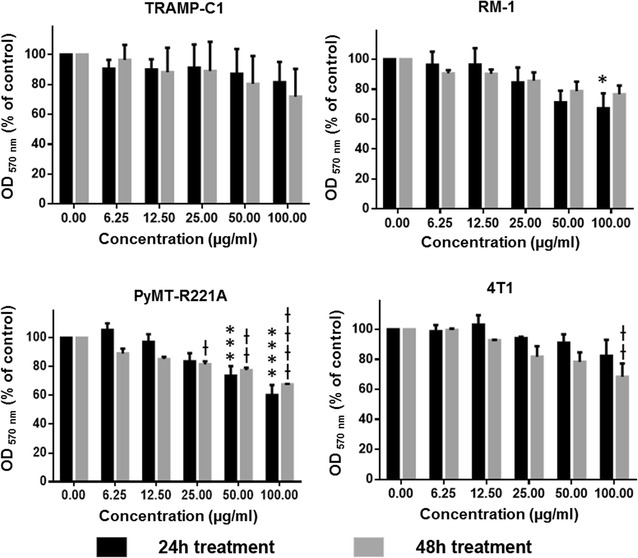
Biocompatibility of YPMS@PpIX@FA measured by MTT assay. Cancer cells were grown and treated with varying concentration of nanoparticles for 24 and 48 h in the absence of light to estimate the dark toxicity. Results are expressed as the mean ± SEM of three individual experiments. *p < 0.05, ***p < 0.001, ****p < 0.0001 vs 24 h control and ^†^p < 0.05, ^††^p < 0.01, ^††††^p < 0.0001 vs 48 h control using a 2-way ANOVA with a Tukey posttest

To ascertain that non-activated YPMS@PpIX@FA nanoparticles are not cytotoxic, which could cause side-effect upon inoculation in vivo, breast and prostate cancer cells were cultured in the presence of increasing concentrations of nanoparticles that were not photo-activated. Cell viability was then measured using an MTT assay. In TRAMP-C1, RM-1 and 4T1 cells, YPMS@PpIX@FA had no or very little effect on cell viability after 24 or 48 h of culture (Fig. 5). PyMT-R221A cells were more sensitive to non-activated YPMS@PpIX@FA nanoparticles, with a 25–40% decrease in viability when using high concentrations of nanoparticles (≥ 50 µg/ml) (Fig. 5). These results show that non-activated nanoparticles have relatively low cytotoxicity when used at concentrations below 25 µg/ml and subsequent experiments were done with concentrations no higher than 25 µg/ml.

**Fig. 6:**
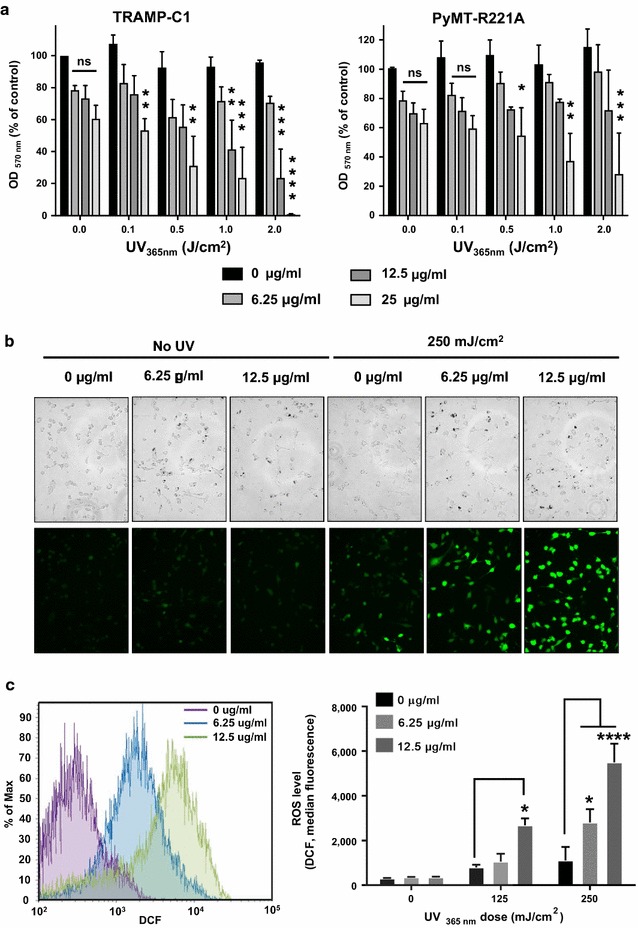
Activation of YPMS@PpIX@FA after UV_365nm_-exposure induces release of ROS and death of cancer cells. **a** For cytotoxicity analysis cells were grown, treated with varying concentration of nanoparticles for 24 h and irradiated with different dose of UV. Toxicity was analyzed by MTT assay 24 h after UV exposure. Generation of cellular ROS on YPMS@PpIX@FA treated TRAMP-C1 cells upon UV irradiation: (**b**) fluorescent microscopy image, (**c**) flow cytometry analysis. Results are expressed as the mean ± SEM of three independent experiments. *ns* not significant, *p < 0.05, **p < 0.01, ***p < 0.001, ****p < 0.0001 using a 2-way ANOVA with a Tukey posttest

We used UV irradiation at 365 nm to activate the PpIX of YPMS@PpIX@FA nanoparticle and mimic PDT. *Folr1*^*hi*^ TRAMP-C1 and PyMT-R221A cells were cultured in the presence of increasing concentrations of YPMS@PpIX@FA for 24 h before UV irradiation. Cell viability was assessed 24 h later using an MTT assay. The doses of UV that were used, 0.1–2 J/cm^2^, had no effect on cancer cell viability (Fig. 6a). Also, photo-activated YPMS@PpIX@FA induced a concentration-dependent decrease in the viability of both cancer cell lines (Fig. 6a). In addition stronger photo-activation of YPMS@PpIX@FA with higher UV irradiation was more efficient at causing cell death in vitro (Fig. 6a). To confirm that the phototoxicity of YPMS@PpIX@FA was due to the functionalized PpIX, PyMT-R221A cells were cultured with non-functionalized YPMS nanoparticles before being exposed to UV irradiation. There was no significant toxicity observed even after exposures of up to 3 J/cm^2^ (Additional file 1: Figure S3a). Since previous reports suggest that silica can interfere with MTT assay, leading to overestimation of cytotoxic effect [43], we tested the effect of photo-activated YPMS@PpIX@FA on PyMT-R221A death using a ß-galactosidase release assay. PyMT-R221A cells were transduced to express ß-galactosidase, which is released into the cell culture media upon cell death and can be detected using a ß-Gal assay [44]. In the absence of nanoparticles or UV irradiation little or no ß-galactosidase activity was detected in culture supernatants. However, after UV irradiation, there was an increase of ß-galactosidase activity confirming that YPMS@PpIX@FA nanoparticles induce a dose-dependent increase in cancer cell death (Additional file 1: Figure S3b). In PDT, cancer cell cytotoxicity is due to the generation of ROS after activation of the PS. To confirm the ability of YPMS@PpIX@FA to generate ROS, TRAMP-C1 and PyMT-R221A were cultured with functionalized nanoparticles before UV irradiation and ROS were detected using DCFDA that is oxidized by ROS into a green fluorescent product (DCF). Using fluorescent microscopy, we observed an increase of green fluorescence in prostate and breast cancer cells after photo-activation of YPMS@PpIX@FA (Fig. 6b and Additional file 1: Figure S4a). Quantification of ROS generation by flow cytometry confirmed that there was a nanoparticle and UV dose-dependent increase of ROS levels in both cancer cell lines (Fig. 6c and Additional file 1: Figure S4b).

**Fig. 7:**
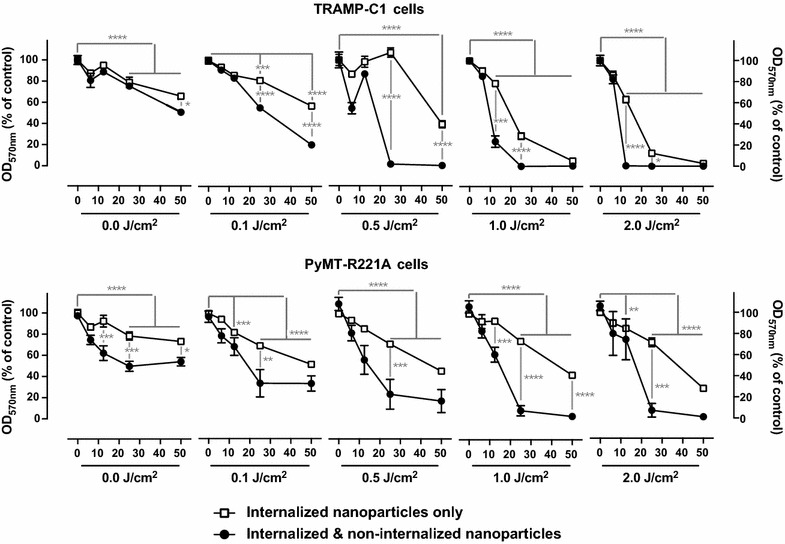
Photoactivation of internalized nanoparticles only decreases breast and prostate cancer cell viability in vitro. TRAMP-C1 and PyMT-R221A cells were grown, treated with varying concentration of nanoparticles for 24 h. Cell monolayers were then washed to keep only the internalized nanoparticles (empty square) in culture or not washed, keeping all nanoparticles (filled circle), internalized and non-internalized in culture. Culture plates were then irradiated with different dose of UV (0–2 J/cm^2^). Toxicity was analyzed by MTT assay 24 h after UV exposure. Results are expressed as the mean ± SEM. *p < 0.05, **p < 0.01, ***p < 0.001, ****p < 0.0001 using a 2-way ANOVA with a Sidak posttest

The decrease of cell viability measured (Fig. 6a) was the consequence of nanoparticles both internalized by the cells and the ones remaining in the media. In patients, it is likely that non-internalized ones would be cleared out and that only internalized nanoparticles would remain to be activated. To reproduce such conditions, after 24 h of treatment, TRAMP-C1 and PyMT-R221A cells were washed so that only internalized nanoparticles remained or not washed so that all nanoparticles remained. Cells were then exposed to UV and viability was measured 24 h later. When all the nanoparticles, internalized and non-internalized ones, were activated, cells were killed in a UV- and nanoparticle-dose dependent matter as previously (Figs. 6a, 7). When only internalized nanoparticles remained and were activated, they were still able to induce a UV- and nanoparticle-dose dependent decrease of cell viability (Fig. 7). Although the effect of internalized nanoparticles only was significantly lower than the effect of all nanoparticles (Fig. 7).

Overall these results demonstrated that the PS PpIX retained its activity after chemical conjugation in YPMS@PpIX@FA nanoparticles, and was able to produce ROS and to cause cancer cell death in vitro.

### Biocompatibility of YPMS@PpIX@FA nanoparticles in vivo

**Fig. 8:**
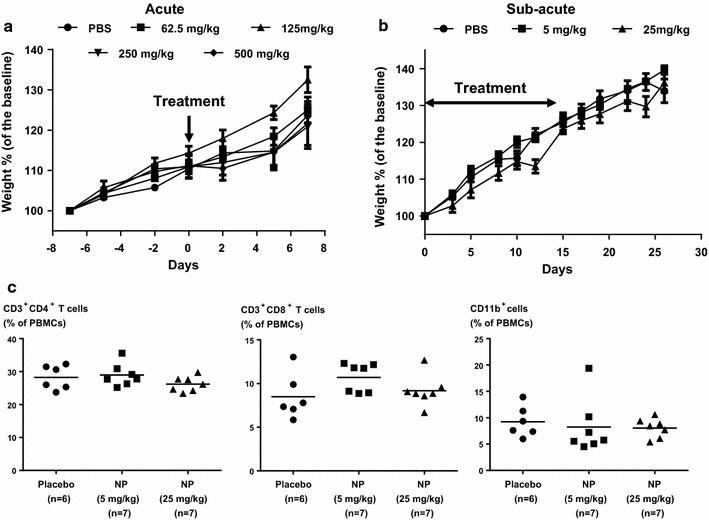
In vivo toxicity evaluation of YPMS@PpIX@FA. For acute toxicity, 25 CD-1 mice were i.v. injected with the indicated doses of nanoparticles (n = 5 per group).** a** Percent change of mouse body weight. The results are shown as mean values ± SEM. Differences were tested using a 2-way ANOVA. For sub-acute toxicity, three groups of mice were i.v. injected with the indicated doses of nanoparticles (n = 7 per group) or PBS (control group, n = 6), thrice a week for 2 weeks. After last injection, blood was collected from the retro-orbital sinus of mice and T cell subsets were characterized by flow cytometry for CD3, CD4, CD8 and CD11b expression.** b** Percent change of mouse body weight. The results are shown as mean values ± SEM. Differences were tested using a 2-way ANOVA.** c** Effect of multiple doses of NP on the circulating levels of CD4^+^ and CD8^+^ T cells and myeloid cells. Results are represented as scatter plots and the bars indicate the average. Differences were tested using a 1-way ANOVA with Tukey posttest

The biocompatibility of YPMS@PpIX@FA nanoparticles was tested in male CD-1 mice in an acute toxicity setting or sub-acute toxicity setting. For acute toxicity, mice received a single intravenous dose of nanoparticles (from 62.5 to 500 mg/kg) or PBS, injected in the tail vein. Inoculation of PBS or 62.5 or 125 mg/kg of YPMS@PpIX@FA nanoparticles did not cause the death of any the treated mice. Doses of 250 or 500 mg/kg resulted in the death of 20% of the mice (1 out 5), immediately after the inoculation and all of the remaining mice survived during the week of observation afterward. To assess overall health, we measured the weight of the mice as well as their food and water consumption for a week before and after the inoculation. No significant difference was observed between the weight of the mice receiving YPMS@PpIX@FA nanoparticles and the mice receiving a placebo (PBS) (Fig. 8a), and there was no apparent effect on food and water consumption (Additional file 1: Figure S5). These results suggest that the LD_50_ of YPMS@PpIX@FA nanoparticles is above 500 mg/kg.

**Fig. 9:**
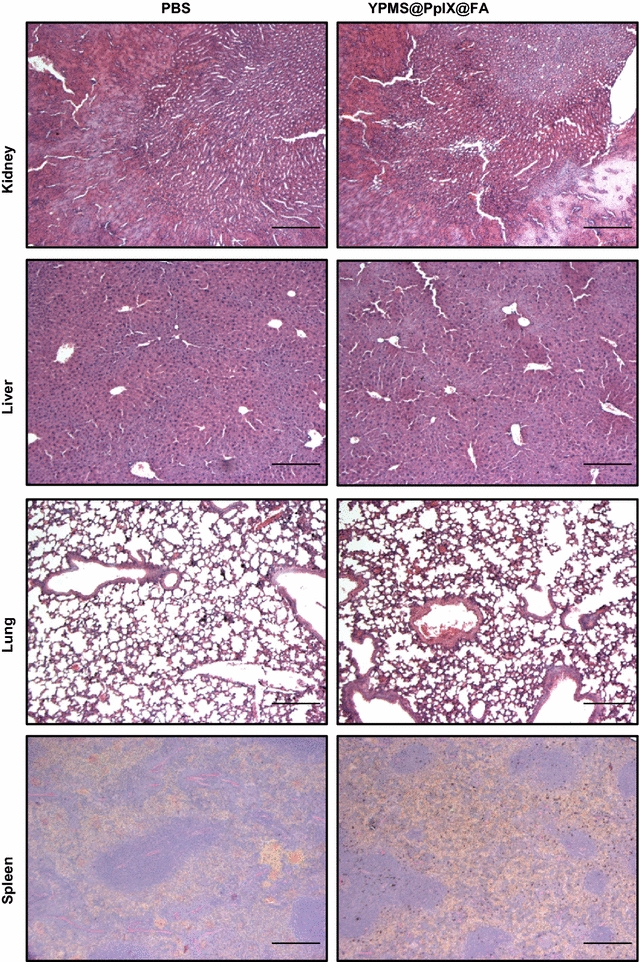
Representative tissue sections of kidney, liver, lung and spleen from mice treated with PBS or nanoparticles (25 mg/kg), stained with hematoxylin and eosin. The scale bar represents 200 μm

For sub-acute toxicity, mice received repeated intravenous inoculation of YPMS@PpIX@FA nanoparticles (5 or 25 mg/kg, 3 times per week) or PBS, during 2 weeks and were then further observed during 2 weeks after cessation of treatment. None of the mice died due to the treatment with YPMS@PpIX@FA nanoparticles over the course of the experiment. In addition, there was no effect of the nanoparticle treatment on mouse weight or food and water consumption when compared to PBS-treated mice (Fig. 8b and Additional file 1: Figure S5). To assess whether the YPMS@PpIX@FA nanoparticles had an effect on the adaptive part of the immune system, mouse peripheral blood was collected after 2 weeks of treatment. We assessed by flow cytometry the amount of circulating helper T cells that are essential for the coordination and activation of the different cells of the immune system and identified by the expression of the protein CD3 and CD4, as well as cytotoxic T cells, essential for elimination of infected or damaged cells identified by the expression of CD3 and CD8. To assess whether the YPMS@PpIX@FA nanoparticles had an effect on the innate immune system, we also measured the circulating levels of myeloid cells that include dendritic cells, monocytes, and macrophages, identified by the expression of CD11b. Treatment with YPMS@PpIX@FA nanoparticles at 5 or 25 mg/kg had no significant effect on the levels of circulating T cells and myeloid cells when compared to mice receiving PBS (Fig. 8c). Also treatment with the nanoparticles did not have any significant effect on the levels CD69 in T cells further showing that YPMS@PpIX@FA did not activate helper and cytotoxic T cells (Additional file 1: Figure S5). To assess damages caused by the nanoparticles in tissues, we performed a histological analysis of the kidney, liver, lung, and spleen of mice. No signs of histopathological abnormalities or lesions were observed in the tissue sections of kidney, liver or lung of mice treated with YPMS@PpIX@FA nanoparticles (25 mg/kg) when compared to PBS-treated mice (Fig. 9). However, observation of sections of spleens from mice treated with YPMS@PpIX@FA (25 mg/kg) revealed the presence of brown-black granular depositions in all the sections observed (Fig. 9 and Additional file 1: Figure S6). In addition, we observed on the spleen section of 1 of the 5 mice analyzed that there was an expansion of the red pulp that is responsible for blood filtration from its antigen. The spleen of the 4 remaining mice did not appear to be different from PBS-treated mice (Fig. 9 and Additional file 1: Figure S6). Overall, these results show that 2 weeks of treatment with YPMS@PpIX@FA nanoparticles were well tolerated by mice and did not show signs of immune response.

## Discussion

The treatment of deep-seated tumors using PDT is largely impeded by the limited penetration of UV–Visible light within organic tissues. The use of X-rays as an alternative source for excitation of PS molecules with the assistance of scintillating nanoparticles as energy transducers has shown encouraging results over the last few years [41]. However, the therapeutic efficacy is still not as satisfactory as conventional PDT due to the low scintillating efficiency of nanoparticles and weak energy transfer from nanoparticles to PS moieties. Moreover, there are a limited number of studies to confirm that PS-nanoscintillator composites are biocompatible, meaning low toxicity to normal tissues as well as limited or absence of effect on the immune response, which is a critical factor for a clinical application. Here, we developed a multifunctionalized, mesoporoussilica-coated nanocomposite, YPMS@PpIX@FA, to be used for targeted PDT of deep-seated tumors. As energy mediator, we used a YP nanoscintillator that emitted photons in the blue and red regions corresponding to the Q-band absorption region of the most commonly used porphyrin-based PS, as similar to most of the lanthanide-doped scintillating nanoparticles [41]. However, production of ROS when energy transfer occurs in the Q-bands is not as efficient as in the Soret region [40]. Our nanoscale YP scintillator also exhibited the strongest emission in the UVA region (320–450 nm) that corresponds to the Soret absorption band of porphyrin-based PS where ROS generation is most efficient. Clement et al. [21] were able to induce a 35% decrease of the viability of pancreatic cancer cells using CeF_3_ nanoparticles that emit in the UVA region corresponding to the Soret band but not in the Q-bands. On the other hand, most of the nanoscintillators developed for X-PDT emit only in the Q-bands, not in the Soret band. In the present study, YP core emitting in both the Soret band and the Q-bands should then be able to activate porphyrin-based PS such as PpIX more efficiently.

To functionalize the nanoparticle with PpIX we coated the YP core with mesoporous silica considering its proven biocompatibility and high cargo-loading ability with hydrophobic PS [25, 45]. In addition, we optimized the coating conditions to obtain a thin layer (14 ± 4 nm) of mesoporous silica ensuring a short distance between the YP core and PpIX, which is critical for efficient energy transfer [46]. Energy transfer between PpIX and the activated YP core was analyzed by photoluminescence analysis. Energy transfer allowed an enhanced release of ROS by PpIX conjugated to YPMS as illustrated by photo-oxygenation of DPBF. Covalent binding of PpIX to the mesoporous silica also improve its overall stability within the nanocomposite, in physiological conditions and prevents its leaking from the pores, to decrease adverse effects in patients [47]. For the nanocomposites to reach all sites of tumor and metastasis in patients with disseminated cancer, local delivery with intratumoral injection, as it is done in most studies [12], is of limited efficiency and systemic administration would be a better strategy. It implies that increasing the affinity of the nanoparticles for cancer cells will eventually reduce the risk of their accumulation in normal cells thereby, preventing the associated side effects. Considering the well-established overexpression of folate receptor α in cancer cells, we chose to functionalize the YPMS@PpIX system with folic acid (FA), which ensured a preferential internalization of the nanoparticle in *Folr1*^*hi*^ breast and prostate cancer cells.

To evaluate the ability of YPMS@PpIX@FA nanoparticles to generate ROS and induce cancer cell death in vitro, we used UV irradiation at 365 nm, which is one of the absorption wavelength of the Soret region. Non-activated YPMS@PpIX@FA nanocomposite did not produce a detectable amount of ROS and had lower cytotoxicity on the breast and prostate cancer cell lines tested for concentrations up to 100 µg/ml. However, upon activation of the nanoparticles with irradiation as low as 125 mJ/cm^2^, generation of ROS was detected. Subsequently, activated YPMS@PpIX@FA nanoparticles induced a significant decrease in breast and prostate cancer cell viability in an irradiation and nanoparticle dose-dependent manner. A ß-galactosidase release assay further confirmed the induction of cancer cell death by ROS. These preliminary results indicate that the activation of the YPMS@PpIX@FA nanoparticles efficiently kills cancer cells thus, suggesting the retained photochemical properties of PS even after covalent conjugation. Further analysis using activation with X-rays would be required to fully assess and confirm the efficacy of YPMS@PpIX@FA for X-PDT in vitro and in vivo.

For clinical application of nanocomposites, it is important to evaluate their biocompatibility in terms of overall toxicity and effect on the immune system. Acute toxicity after inoculation of a single high dose was tested as well as sub-acute toxicity after repeated inoculation of lower doses. Systemic administration via injection in the tail vein of YPMS@PpIX@FAdid not cause the death of mice inoculated with a single dose of up to 125 mg/kg or any detectable changes in their behavior. Similarly, 2 weeks of i.v. treatment with YPMS@PpIX@FA doses of up to 25 mg/kg did not cause the death of any mice, and no adverse events were detected when evaluating weight gain or feeding habits. Also, the histopathological analysis did not show any effect of the YPMS@PpIX@FA nanoparticles in lung, kidney or liver tissues. However, there were some brown/black color granular depositions in the spleens of mice treated with the nanoparticles. It is unclear what this deposits are at the moment. They could be accumulations of nanoparticles in spleen. It was previously found that nanoparticles larger than 150 nm in diameter can accumulate in the spleen [48]. Despite of this deposition, there were no differences in the amount of circulating CD4^+^ and CD8^+^ T cells in mice treated with YPMS@FA@PpIX when compared to vehicle treated mice. Only 1 spleen of the 5 nanoparticle-treated mice analyzed showed signs of red pulp expansion in the spleen. This mouse was having the highest levels of circulating CD4^+^ and CD8^+^ T cells within its group, although their activation was not different according to CD69 levels (data not shown). Despite of that this mouse did not have sign of weight loss or morphological changes in the other tissues analyzed (kidney, lungs and liver). Finally, there was no apparent effect of the nanoparticles on circulating CD11b^+^ myeloid cells among which are dendritic cells, monocytes, and macrophages, as well as helper or cytotoxic T cells in the mice, tested for sub-acute toxicity. This safety evaluation indicates that YPMS@PpIX@FA nanoparticles have therefore a good biocompatibility and do not induce an immune response at the given doses.

## Conclusions

In conclusion, we have synthesized and characterized a mesoporous silica coated YP based nanoparticles, YPMS@PpIX@FA with the well-defined nanostructure, good radiation stopping power and with high luminescence efficiency in the 300–450 nm range corresponding to the maximum absorption of many porphyrin-based photosensitizers, making it optimal for X-PDT. Functionalization with folic acid increased the affinity and uptake of this nanocomposite in *Folr1*^*hi*^ cancer cells in vitro, to reduce the risk of side effect and biocompatibility studies in mice showed little or no toxicity as well as no apparent effect on the immune system of YPMS@PpIX@FA nanocomposite. Photoactivation of these nanoparticles led to efficient ROS generation causing breast and prostate cancer cell death in vitro. More studies are underway to validate the efficiency of X-PDT using YPMS@PpIX@FA in vitro and in vivo. Overall these data suggest that the YPMS system may be a potential platform for X-PDT of deep-seated tumors when functionalized with FA and PpIX, and other targeting agents or PS molecules could be used for functionalization making it a versatile platform for X-PDT.

## Additional file

**Additional file 1.** Figures.

## Abbreviations

APTMS: aminopropyltrimethoxysilane
BET: Brunauer–Emmett–Teller
BJH: Barrerr–Joyner–Halanda
DCFDA: 2′,7′-dichlorofluorescin diacetate
DLS: dynamic light scattering
FA: folic acid
*Folr1*: folate receptor 1
FRET: fluorescence resonance energy transfer
FTIR: Fourier transform infrared spectroscopy
i.v.: intravenously
λ_ex_: excitation wavelength
λ_em_: emission wavelength
LD_50_: median lethal dose
PDT: photodynamic therapy
PL: photoluminescence
PpIX: protoporphyrin IX
PS: photosensitizer
ROS: reactive oxygen species
TEM: transmission electron microscopy
TEOS: tetraethyl orthosilicate
UV: ultraviolet
X-PDT: X-ray mediated PDT
YAG: Y_3_Al_5_O_12_
YP: Y_2.99_Pr_0.01_Al_5_O_12_
YPMS: YP coated with mesoporous silica

## References

[1] Stewart BW, Wild CP (2014). World cancer report 2014.

[2] Dolmans DE, Fukumura D, Jain RK (2003). TIMELINE: photodynamic therapy for cancer. Nat Rev Cancer..

[3] Agostinis P, Berg K, Cengel KA, Foster TH, Girotti AW, Gollnick SO (2011). Photodynamic therapy of cancer: an update. CA Cancer J Clin.

[4] Kachynski AV, Pliss A, Kuzmin AN, Ohulchanskyy TY, Baev A, Qu J (2014). Photodynamic therapy by in situ nonlinear photon conversion. Nat Photonics.

[5] Schmitt J, Heitz V, Sour A, Bolze F, Ftouni H, Nicoud J-F (2015). Diketopyrrolopyrrole–porphyrin conjugates with high two-photon absorption and singlet oxygen generation for two-photon photodynamic therapy. Angew Chem Int Ed..

[6] Yuan A, Wu J, Tang X, Zhao L, Xu F, Hu Y (2013). Application of near-infrared dyes for tumor imaging, photothermal, and photodynamic therapies. J Pharm Sci.

[7] Ntziachristos V, Bremer C, Weissleder R (2003). Fluorescence imaging with near-infrared light: new technological advances that enable in vivo molecular imaging. Eur Radiol.

[8] Seo S-H, Kim B-M, Joe A, Han H-W, Chen X, Cheng Z (2014). NIR-light-induced surface-enhanced Raman scattering for detection and photothermal/photodynamic therapy of cancer cells using methylene blue-embedded gold nanorod@SiO_2_ nanocomposites. Biomaterials.

[9] Chen N-T, Tang K-C, Chung M-F, Cheng S-H, Huang C-M, Chu C-H (2014). Enhanced plasmonic resonance energy transfer in mesoporous silica-encased gold nanorod for two-photon-activated photodynamic therapy. Theranostics..

[10] Wang C, Tao H, Cheng L, Liu Z (2011). Near-infrared light induced in vivo photodynamic therapy of cancer based on upconversion nanoparticles. Biomaterials.

[11] Lucky SS, Muhammad Idris N, Li Z, Huang K, Soo KC, Zhang Y (2015). Titania coated upconversion nanoparticles for near-infrared light triggered photodynamic therapy. ACS Nano.

[12] Hu J, Tang Y, Elmenoufy AH, Xu H, Cheng Z, Yang X (2015). Nanocomposite-based photodynamic therapy strategies for deep tumor treatment. Small.

[13] Chen W, Zhang J (2006). Using nanoparticles to enable simultaneous radiation and photodynamic therapies for cancer treatment. J Nanosci Nanotechnol.

[14] Liu Y, Chen W, Wang S, Alan GJ (2008). Investigation of water-soluble X-ray luminescence nanoparticles for photodynamic activation. Appl Phys Lett.

[15] Bulin A-L, Truillet C, Chouikrat R, Lux F, Frochot C, Amans D (2013). X-ray-induced singlet oxygen activation with nanoscintillator-coupled porphyrins. J Phys Chem C.

[16] Kitis G, Furetta C, Prokic M, Prokic V (2000). Kinetic parameters of some tissue equivalent thermoluminescence materials. J Phys D Appl Phys.

[17] Ma L, Zou X, Bui B, Chen W, Song KH, Solberg T (2014). X-ray excited ZnS:Cu, Co afterglow nanoparticles for photodynamic activation. Appl Phys Lett.

[18] Chen H, Wang GD, Chuang Y-JJ, Zhen Z, Chen X, Biddinger P (2015). Nanoscintillator-mediated X-ray inducible photodynamic therapy for in vivo cancer treatment. Nano Lett.

[19] Kaščáková S, Giuliani A, Lacerda S, Pallier A, Mercère P, Tóth É (2015). X-ray-induced radiophotodynamic therapy (RPDT) using lanthanide micelles: beyond depth limitations. Nano Res..

[20] Liu Y, Zhang Y, Wang S, Pope C, Chen W, Liu Y (2013). Optical behaviors of ZnO-porphyrin conjugates and their potential applications for cancer treatment optical behaviors of ZnO-porphyrin conjugates and their potential. Appl Phys Lett.

[21] Clement S, Deng W, Camilleri E, Wilson BC, Goldys EM (2016). X-ray induced singlet oxygen generation by nanoparticle-photosensitizer conjugates for photodynamic therapy: determination of singlet oxygen quantum yield. Sci Rep..

[22] Jung JY, Hirata GA, Gundiah G, Derenzo S, Wrasidlo W, Kesari S (2014). Identification and development of nanoscintillators for biotechnology applications. J Lumin.

[23] Dong B, Wang J, Sun J, Xu S, Bai X, Jiang Z (2012). Non-photobleaching YAG: Ce nanoparticles for optical imaging with blue excitation. RSC Adv..

[24] Asakura R, Isobe T, Kurokawa K, Aizawa H, Ohkubo M (2006). Tagging of avidin immobilized beads with biotinylated YAG:Ce^3+^ nanocrystal phosphor. Anal Bioanal Chem.

[25] Tu H-L, Lin Y-S, Lin H-Y, Hung Y, Lo L-W, Chen Y-F (2009). In vitro studies of functionalized mesoporous silica nanoparticles for photodynamic therapy. Adv Mater.

[26] Derycke ASL, De Witte PAM (2004). Liposomes for photodynamic therapy. Adv Drug Deliv Rev.

[27] Van Nostrum CF (2004). Polymeric micelles to deliver photosensitizers for photodynamic therapy. Adv Drug Deliv Rev.

[28] Wang XL, Zeng Y, Zheng YZ, Chen JF, Tao X, Wang LX (2011). Rose bengal-grafted biodegradable microcapsules: singlet-oxygen generation and cancer-cell incapacitation. Chem A Eur J..

[29] Vivero-Escoto JL, Vega DL (2014). Stimuli-responsive protoporphyrin IX silica-based nanoparticles for photodynamic therapy in vitro. RSC Adv..

[30] Zhao ZX, Huang YZ, Shi SG, Tang SH, Li DH, Chen XL (2014). Cancer therapy improvement with mesoporous silica nanoparticles combining photodynamic and photothermal therapy. Nanotechnology..

[31] Couleaud P, Morosini V, Frochot C, Richeter S, Raehm L, Durand J-O (2010). Silica-based nanoparticles for photodynamic therapy applications. Nanoscale..

[32] Xia W, Low PS (2010). Folate-targeted therapies for cancer. J Med Chem.

[33] Sengar P, Hirata GAA, Farias MH, Castillón F (2016). Morphological optimization and (3-aminopropyl) trimethoxy silane surface modification of Y_3_Al_5_O_12_: Pr nanoscintillator for biomedical applications. Mater Res Bull.

[34] Stöber W, Fink A, Bohn E (1968). Controlled growth of monodisperse silica spheres in the micron size range. J Colloid Interface Sci.

[35] Ohyashiki T, Nunomura M, Katoh T (1999). Detection of superoxide anion radical in phospholipid liposomal membrane by fluorescence quenching method using 1,3-diphenylisobenzofuran. BBA Biomembr.

[36] nanoComposix. Uv/Vis/Ir spectroscopy analysis of nanoparticles. https://nanocomposix.com/products/uv-visible-nanoparticle-analysis. Accessed 20 Jan 2018.

[37] Sancak Y, Peterson TR, Shaul YD, Lindquist RA, Thoreen CC, Bar-Peled L (2008). The rag GTPases bind raptor and mediate amino acid signaling to mTORC1. Science.

[38] Untergasser A, Nijveen H, Rao X, Bisseling T, Geurts R, Leunissen JA (2007). Primer3Plus, an enhanced web interface to Primer3. Nucleic Acids Res.

[39] Servicio Nacional de Sanidad, Inocuidad y Calidad Agroalimentaria. NORMA Oficial Mexicana NOM-062-ZOO-1999, Especificaciones técnicas para la producción, cuidado y uso de los animales de laboratorio. 1999. https://www.gob.mx/senasica/documentos/nom-062-zoo-1999. Accessed 28 July 2016.

[40] Buchczyk DP, Klotz L-O, Lang K, Fritsch C, Sies H (2001). High efficiency of 5-aminolevulinate-photodynamic treatment using UVA irradiation. Carcinogenesis.

[41] Kamkaew A, Chen F, Zhan Y, Majewski RL, Cai W (2016). Scintillating nanoparticles as energy mediators for enhanced photodynamic therapy. ACS Nano.

[42] Zwicke GL, Mansoori GA, Jeffery CJ (2012). Utilizing the folate receptor for active targeting of cancer nanotherapeutics. Nano Rev..

[43] Fisichella M, Dabboue H, Bhattacharyya S, Saboungi M-LL, Salvetat J-PP, Hevor T (2009). Mesoporous silica nanoparticles enhance MTT formazan exocytosis in HeLa cells and astrocytes. Toxicol In Vitr.

[44] Debacq-Chainiaux F, Erusalimsky JD, Campisi J, Toussaint O (2009). Protocols to detect senescence-associated beta-galactosidase (SA-βgal) activity, a biomarker of senescent cells in culture and in vivo. Nat Protoc.

[45] Liu T, Li L, Teng X, Huang X, Liu H, Chen D (2011). Single and repeated dose toxicity of mesoporous hollow silica nanoparticles in intravenously exposed mice. Biomaterials.

[46] Chen H, Puhl HL, Koushik SV, Vogel SS, Ikeda SR (2006). Measurement of FRET efficiency and ratio of donor to acceptor concentration in living cells. Biophys J.

[47] Wang Z, Hong X, Zong S, Tang C, Cui Y, Zheng Q (2015). BODIPY-doped silica nanoparticles with reduced dye leakage and enhanced singlet oxygen generation. Sci Rep..

[48] Blanco E, Shen H, Ferrari M (2015). Principles of nanoparticle design for overcoming biological barriers to drug delivery Nat. Biotech..

