# Supplementary material for: Development of a functionalized UV-emitting nanocomposite for the treatment of cancer using indirect photodynamic therapy
Source: J Nanobiotechnology. 2018 Feb 27;16:19. doi: 10.1186/s12951-018-0344-3 (PMC5827996; doi:10.1186/s12951-018-0344-3)
Supplement: Supplementary file 1 — Additional file 1. Figures. [file 12951_2018_344_MOESM1_ESM.pdf]

Development of a functionalized UV-emitting nanocomposite for the treatment of cancer using indirect photodynamic therapy

Prakhar Sengar[1,2,3], Patricia Juárez[1], Andrea Verdugo-Meza[1], Danna L. Arellano[1], Akhil Jain[1,2,3], Kanchan Chauhan[2], Gustavo A. Hirata[2] and Pierrick GJ. Fournier[1].

Affiliation of authors:

[1] Biomedical Innovation Department - Centro de Investigación Científica y de Educación Superior de Ensenada, Baja California, (CICESE), Ensenada, Baja California, México.

[2] Centro de Nanociencias y Nanotecnología (CNyN) - Universidad Nacional Autónoma de México (UNAM) – Ensenada, Baja California, México.

[3] Posgrado en Física de Materiales - Centro de Investigación Científica y de Educación Superior de Ensenada, Baja California, (CICESE), Ensenada, Baja California, México.

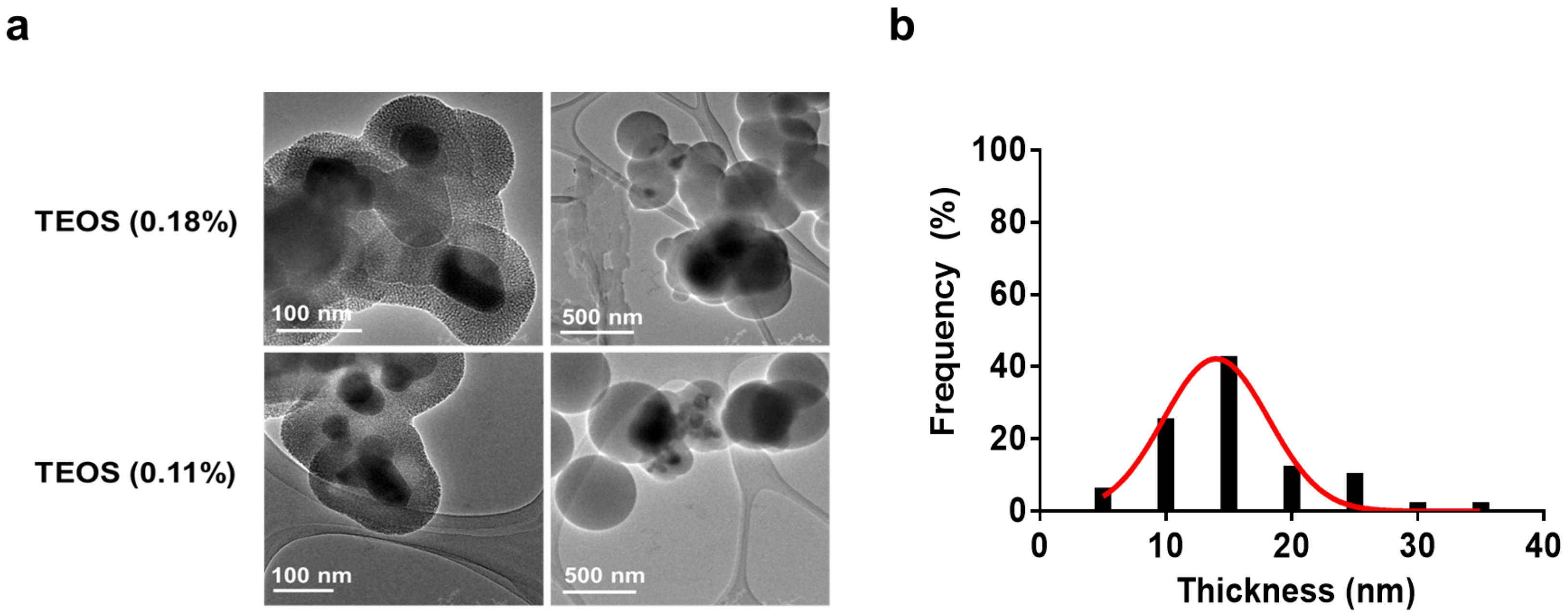

Figure S1. (a) TEM micrographs of mesoporous silica coated YAG:Pr synthesized using 0.18% or0.11% of TEOS. **(b)** Size distribution histogram of mesoporous silica coating on YPMS.

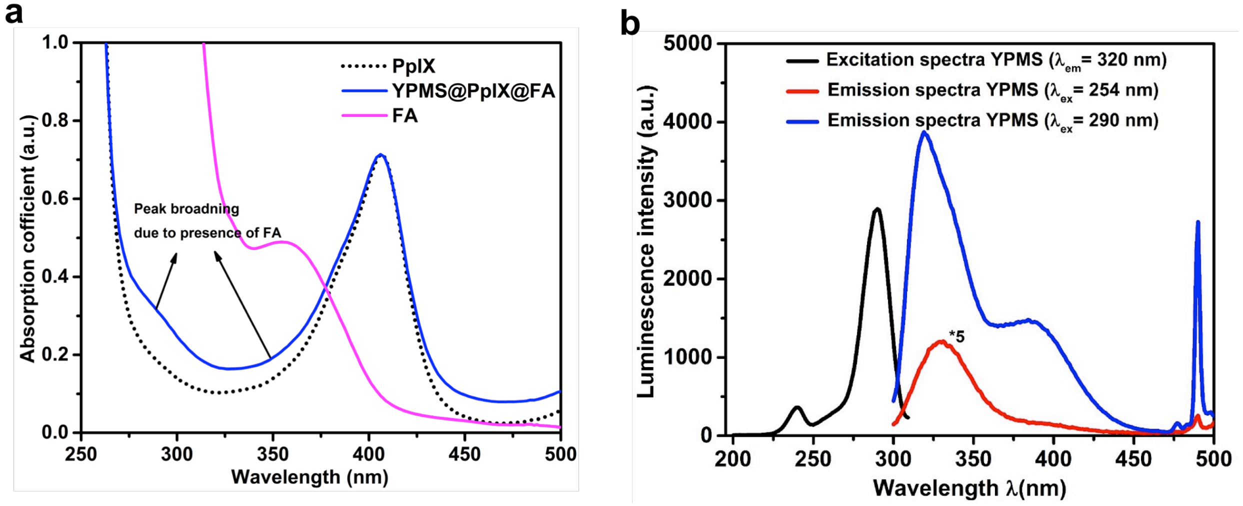

Figure S2. **(a)** UV-Vis spectra of PpIX, FA and YPMS@PpIX@FA nanoparticles. **(b)** Photoluminescence excitation and emission spectra of YPMS. In the red curve, *5 indicates the 5-fold increase in the emission spectrum from the initial value.

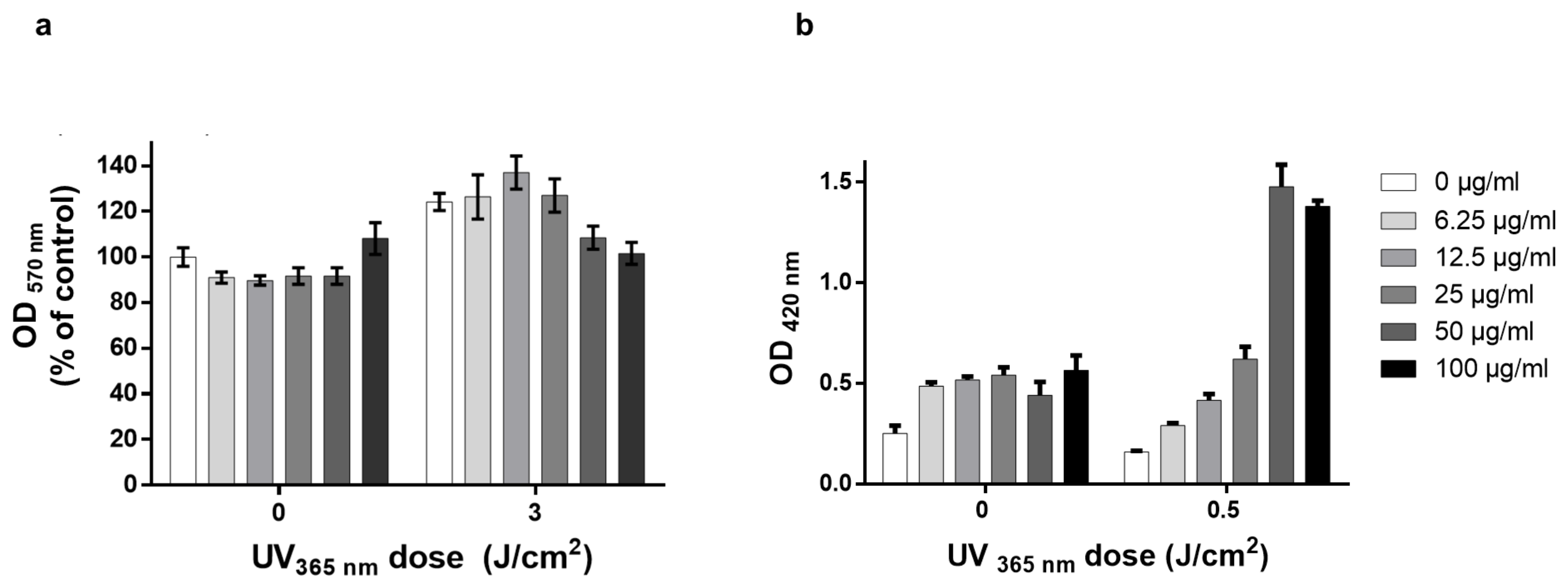

Figure S3. Cytotoxicity analysis of YPMS and YPMS@PpIX@FA nanoparticles. **(a)** UV exposure on YPMS treated PyMT-R221A cells had no significant effect on cell viability. For cytotoxicity analysis cells were grown, treated with increasing concentrations of nanoparticles for 24 h and irradiated with 3 J/cm^2 UV dose. Cell viability was analyzed using an MTT assay, 24 h after the UV exposure. **(b)** ß-Galactosidase release assay of YPMS@PpIX@FA on PyMT-ßGal cells.

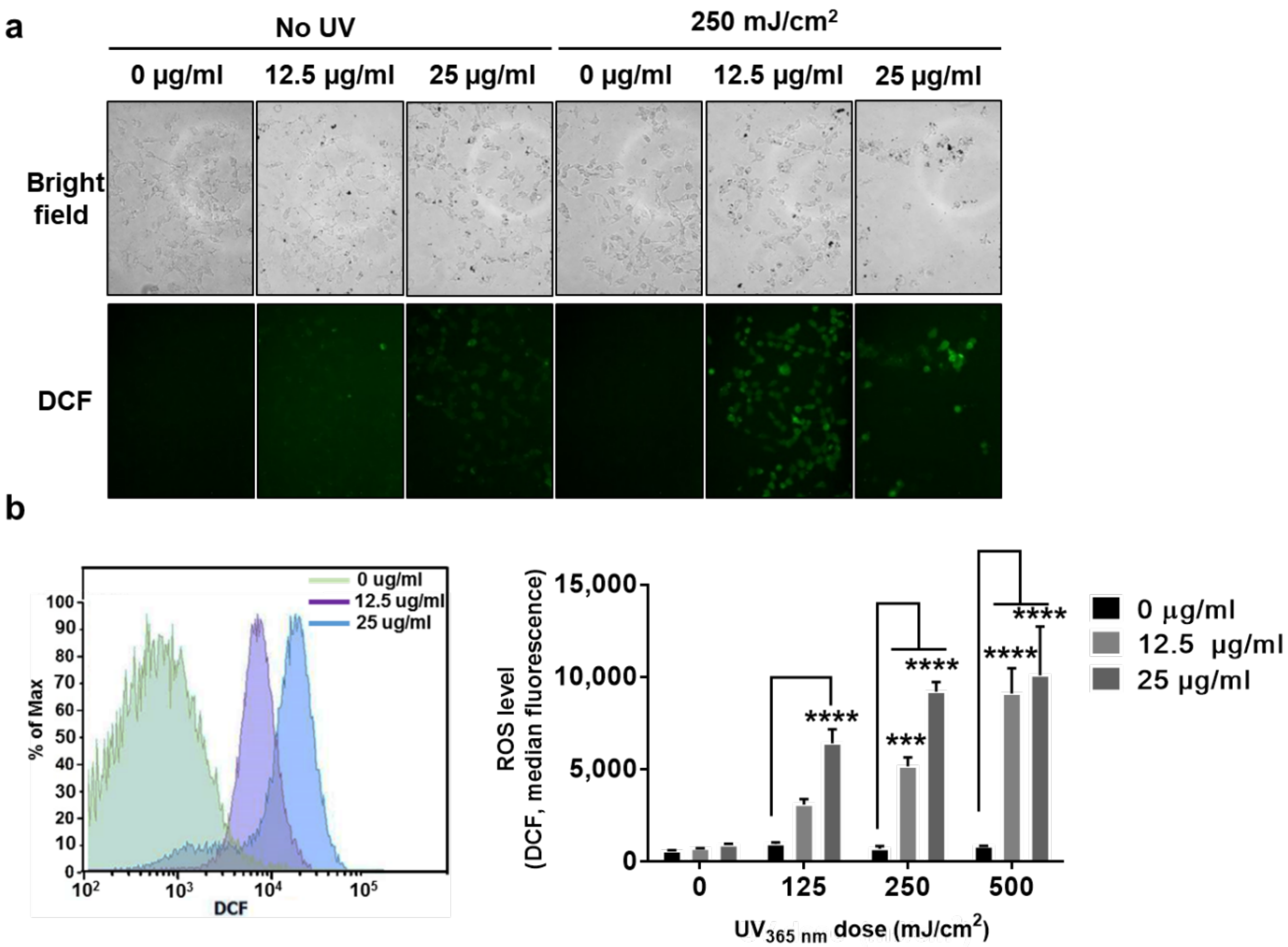

Figure S4. Generation of cellular ROS on YPMS@PpIX@FA treated PyMT cells upon UV$_{(365\ nm)}$ irradiation. (a) Fluorescent microscopy image. **(b)** Flow cytometry analysis. Results are expressed as the mean ± SEM of three independent experiments. *p < 0.05, **p < 0.01, ***p < 0.001, ****p < 0.0001 using a 2-way ANOVA with Tukey posttest.

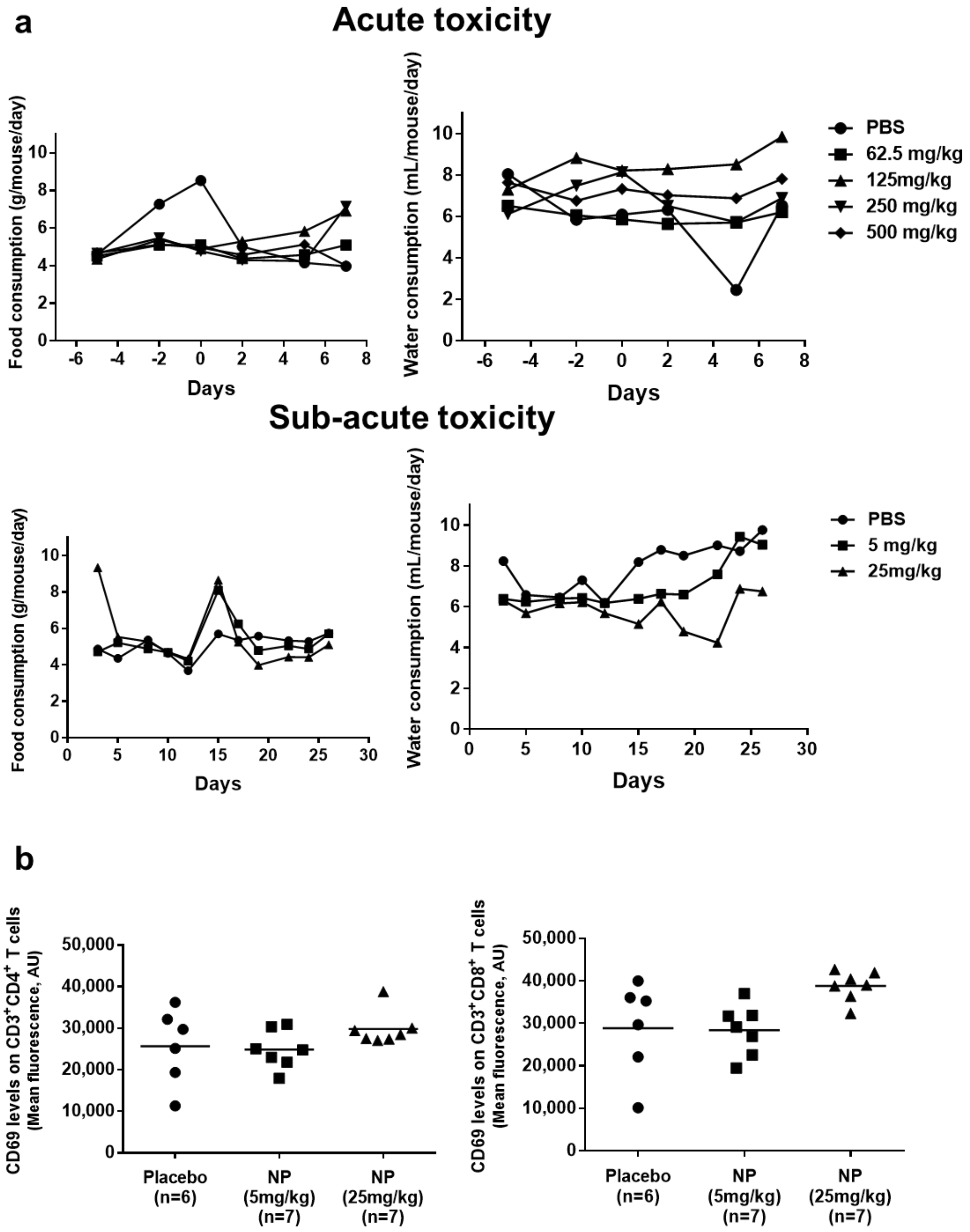

Figure S5. Effect of treatment with YPMS@PpIX@FA nanoparticles on the feeding of mice and T cell activation. (a) Food and water consumption for acute and sub-acute toxicity of YPMS@PpIX@FA. (b) Effect of multiple dose of NP on CD69 expression in T lymphocytes. Results were analyzed using a 1-way ANOVA with Tukey posttest.

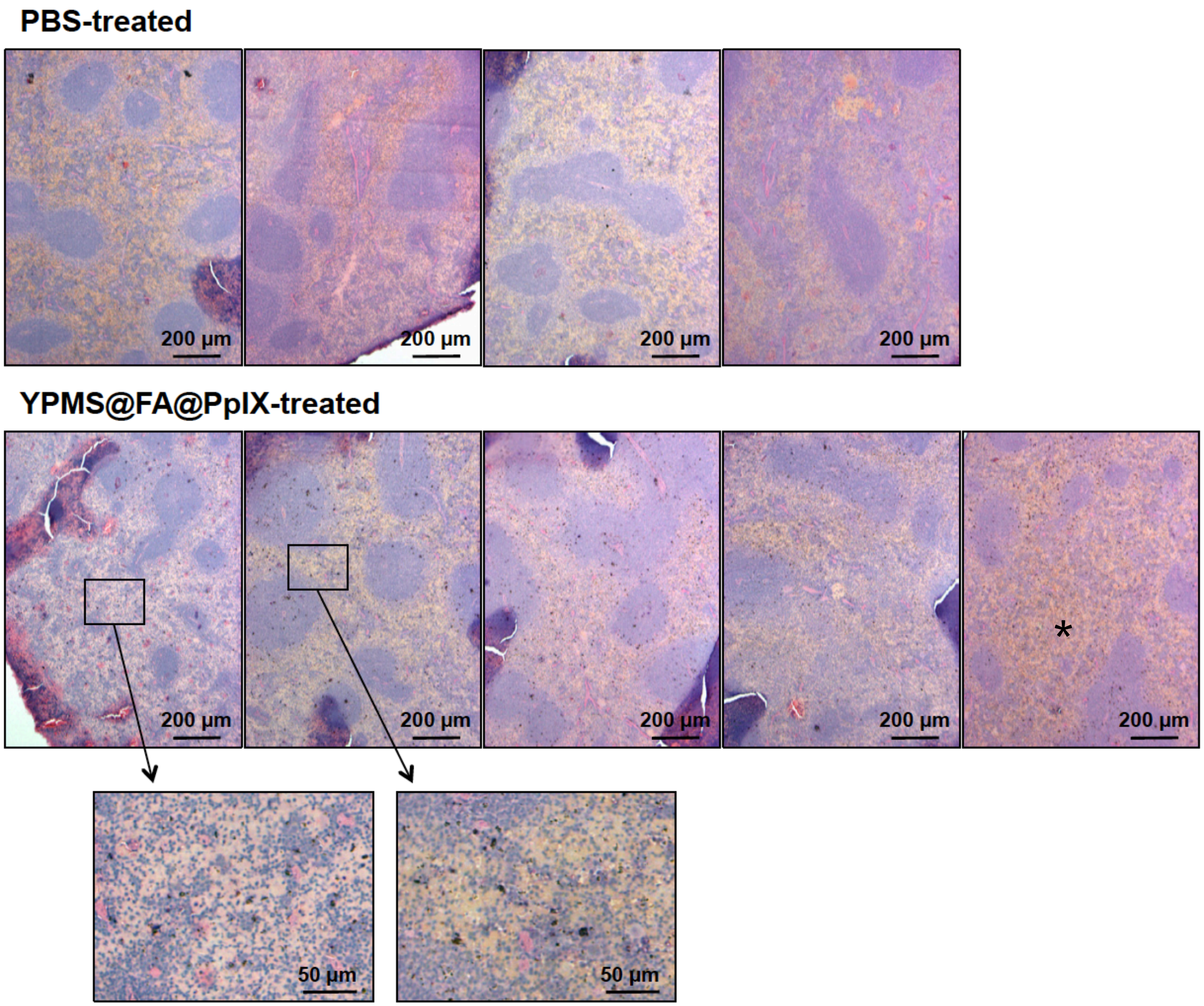

Figure S6. Tissue sections of spleens of mice treated with PBS (n = 4) or YPM@FA@PpIX nanoparticles (25 mg/kg) (n = 5), stained with hematoxylin and eosin. All the spleens of the mice treated with YPMS@FA@PpIX showed the presence of brown/black deposits (see higher magnification pictures and 1 of these mice showed signs of red pulp expansion (indicated by *).
